# Insights into the Chemistry of Non-Enzymatic Browning Reactions in Different Ribose-Amino Acid Model Systems

**DOI:** 10.1038/s41598-018-34335-5

**Published:** 2018-11-15

**Authors:** Daniel Hemmler, Chloé Roullier-Gall, James W. Marshall, Michael Rychlik, Andrew J. Taylor, Philippe Schmitt-Kopplin

**Affiliations:** 1 Comprehensive Foodomics Platform, Analytical Food Chemistry, Technical University Munich, Alte Akademie 10, 85354 Freising, Germany; 2 Research Unit Analytical BioGeoChemistry (BGC), Helmholtz Zentrum München, Ingolstädter Landstrasse 1, 85764 Neuherberg, Germany; 3 The Waltham Centre for Pet Nutrition, Mars Petcare UK, Waltham-on-the-Wolds, Leicestershire LE14 4RT United Kingdom

## Abstract

Reactions between sugars and amino acids in the Maillard reaction produce a multitude of compounds through interconnected chemical pathways. The course of the pathways changes depending on the nature of the amino acids and sugars as well as the processing conditions (*e.g*. temperature, water activity). Some partial pathways have been elucidated using labelled precursors but the process is very time intensive. Here, we use rapid, non-targeted analysis with Fourier transform ion cyclotron resonance mass spectrometry (FT-ICR-MS) to deliver the molecular formulae and ion intensities of the compounds generated from reaction of four amino acids with ribose (10 h at 100 °C) to study the effect of amino acid side chains on the reaction pathways. Using van Krevelen diagrams, known chemical changes during the reaction (*e.g*. dehydration or decarboxylation) can be studied. Comparison of the data from the four amino acids studied, showed a common pathway, which involved 73 Maillard reaction products (MRPs) where the differences were due only to the nature of the amino acid side chain. From the more than 1400 different molecular formulae found, pathways unique to the amino acids were also identified and the order of reactivity was lysine >cysteine >isoleucine ≈ glycine. While unequivocal identification of the compounds cannot be achieved with FT-ICR-MS, applying known chemical transformations found in the Maillard reaction, not only identifies new and known pathways, but also integrates the MRPs into a general Maillard reaction scheme that better represents the totality of the Maillard reaction.

## Introduction

Non-enzymatic browning between reducing carbohydrates and amines, also known as the Maillard reaction (MR), is of crucial importance in food science where it significantly contributes to taste, aroma and color^1^. Additionally, it is now established that the MR takes place *in vivo* under physiological conditions, where non-enzymatic reactions between carbohydrates and proteins lead to irreversible protein modifications associated with a wide range of diseases, such as *diabetes mellitus*^1–3^. The initial condensation between an amine compound (*e.g*. amino acid) and carbonyls leads to the relatively stable Amadori rearrangement product (ARP). Subsequent breakdown of the ARP (intermediate phase) initiates a flood of chemical reactions continuously producing new intermediates which are fed into the Maillard reaction pool. Many of the breakdown intermediates produced are highly reactive, such as reductones and other (di)carbonyl compounds^4^, which then may react to form new Maillard reaction products (MRPs), thus increasing the chemodiversity exponentially. Among many other factors, type and concentration of precursors, temperature, pH and time have a major impact on the type of reactions and intermediates as well as the end-products produced. This makes the MR certainly one of the most complex reaction “collectives” which is able to produce thousands of distinct chemical compounds from only a few initial precursors.

To date, detailed knowledge of reaction mechanisms has been achieved for the some specific steps of the MR, mainly from studies of sugar-amino acid model systems but no overall view of the reaction pathways has been published. By means of non-targeted GC-MS methods, many volatile, often flavour-active molecules or precursors thereof, have been identified, and important formation pathways proposed^5,6^. By comparison, non-volatiles of the intermediate phase are still unknown to a large extent. The high diversity in chemical properties makes a simultaneous analysis especially challenging^7,8^. Although several reported methods have successfully separated and detected multiple reaction products, non-targeted analysis exploring the non-volatiles, produced throughout the course of the MR, are rare^9^. The large number of reaction products, reactive intermediates and transition species, often having very similar retention properties, do not allow sufficient isolation and structure elucidation of all of the compounds involved in the MR. Hence, most of the described analytical approaches focus on quantification of selected target molecules. For example, Davidek *et al*. proposed a derivatisation-free anion exchange chromatography method for the simultaneous quantitation of sugar and amino acid precursors together with the Amadori rearrangement product (ARP) and three cyclic intermediates^9^. More recently, Katayama *et al*. developed an LC-MS/MS method for quantification of twenty fructose-derived ARPs^10^.

Although mass spectrometry alone cannot provide sufficient structural information for unequivocal identification, it is an irreplaceable tool for the holistic analysis of complex samples on a molecular level. In recent years, ultrahigh-resolution Fourier transform ion cyclotron resonance mass spectrometry (FT-ICR-MS) has prevailed as a method of choice in the compositional characterization of utmost complex samples in many scientific disciplines^11–13^. We have recently shown that direct-infusion FT-ICR-MS provides deep insights into initial and intermediate MRPs produced in a ribose-glycine Maillard model system. We found more than 300 distinct elemental compositions, produced in the ribose-glycine Maillard reaction cascade^14^. In the present study, we extend our experiment using multiple amino acid precursors. The aim is to demonstrate the chemodiversity in the molecular characteristics among different model systems on the level of accurate molecular formulae in order to generate new hypotheses, which could help to improve the understanding of the chemistry of MRPs and their formation processes.

## Results and Discussion

### Reaction monitoring by direct-infusion FT-ICR mass spectrometry

Four different amino acids (glycine, isoleucine, lysine, and cysteine) were reacted with ribose in equimolar (0.1 M) mixtures at 100 °C. We monitored the formation of intermediates and reaction products by direct-infusion FT-ICR-MS after a reaction time of two, four, six, and ten hours. In this fundamental proof-of-principle study, we used a well-known but uncontrolled reaction system (unbuffered solutions). The amino acids were chosen to cover a wide range of physicochemical properties. Ribose was selected as the major carbohydrate precursor to study because of its high reactivity among the common pentoses and hexoses^15^.

Here, we focus on results obtained after negative electrospray (ESI(−)) ionization which allows detection of polar and oxygen-rich MRPs of the initial and intermediate phase of the MR. Hundreds of distinct ion signals could be recorded in each model system (Fig. S1a). Comparing the four amino acids, lysine showed a considerably higher signal density after ten hours compared to the other amino acids. Most of the reaction products were found in a mass range between *m/z* 100–600. While the high resolving power (400 000 at *m/z* 300) allowed an unambiguous differentiation of the recorded ion signals, high mass accuracy and precision allowed us to assign detected *m/z*-values to their unambiguous molecular formulae. If only C_c_H_h_O_o_ element compositions are considered it would be possible, with the achieved mass accuracy, to accurately assign molecular formulae using only the exact ion masses. However, when non-oxygen heteroatoms, such as nitrogen or sulphur, are also taken into account, the number of possible solutions increases dramatically with the number of heteroatoms and ion mass^16^. Hence, we used a combination of compositional network-based formulae annotation and isotopic fine structure validation (Fig. S1b) in order to eliminate false assignments^17–19^. After ten hours, we found in total 1493 distinct molecular formulae among the four investigated model systems. More than 90% of all features were found within a maximum error of ± 0.2 ppm (99% with maximum error of ± 0.5 ppm, Fig. S1c).

Here, we report only features, which were found in all three replicates. This guarantees highest accuracy and reproducibility of the discussed compounds. Spectra were always dominated by a few high-intensity principal components while the majority of produced compounds were found with considerably lower peak intensities (Fig. S1c). Overall, we observed analytes in a range covering approximately four orders of magnitude in peak intensity. This intensity range, covered by the ICR mass analyser, can also be interpreted as an approximation for the relative concentration range in which compounds occur in the MR. The similarity in chemical properties together with the complexity and high dynamic range in concentrations makes a simultaneous or comprehensive analysis with conventional analytical approaches, such as LC-MS, very challenging^7,8^.

### Formation of reaction products monitored over time

**Figure 1:**
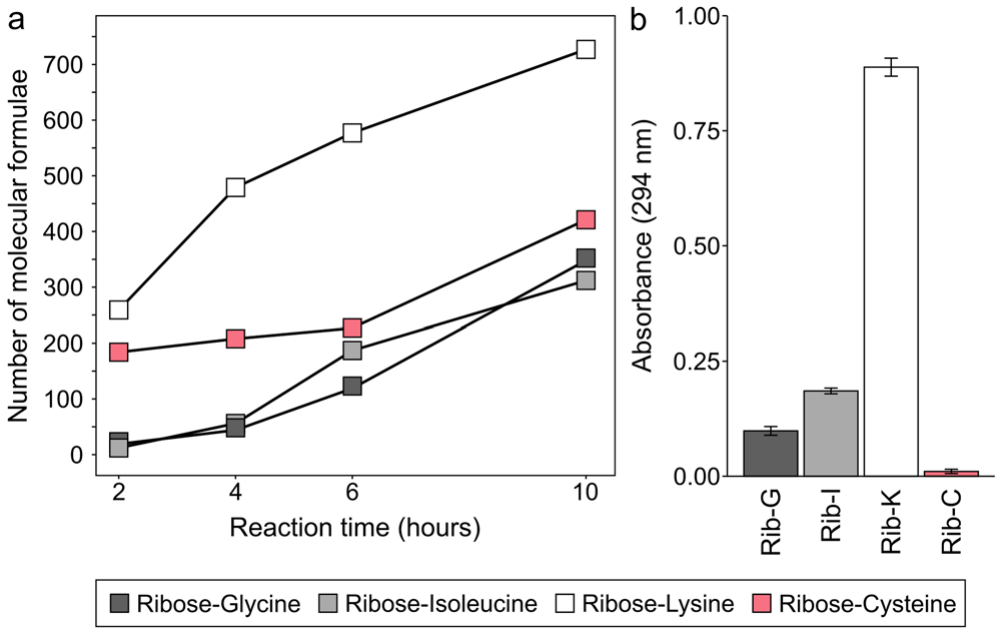
Formation of MRPs and UV absorbing products. (**a)** Number of MRPs produced in four different ribose-amino acid model systems heated for two, four, six, and ten hours (100 °C). (**b)** Absorbance at 294 nm of ribose-amino acid models heated for ten hours (100 °C). Error bars indicate the standard deviation of the mean absorbance value (n = 3).

With the help of blank samples (ribose and amino acids heated alone, respectively), the recorded reaction products were classified into thermally synthesized MRPs, sugar and amino acid degradation products according to the classification approach suggested by Yaylayan^20^ and as recently described^14^. Most reaction products could be clearly assigned to MRPs, which require for their formation the presence of both, an amino acid and a sugar precursor. Among the tested amino acids, lysine produced most MRPs. After ten hours of thermal treatment, we could detect more than 700 different molecular formulae assigned to MRPs. By comparison, for glycine, isoleucine and cysteine we found 300–400 MRPs, respectively. The order of MRPs produced after ten hours was lysine >cysteine >isoleucine ≈ glycine (Fig. 1a). Lysine was also shown in many other studies to be the most reactive amino acid in Maillard model systems^21–23^ and the key contributor to MRPs produced through protein glycation. Interestingly, cysteine and lysine both showed a high number of produced MRPs after heating the samples for only two hours. Compared to glycine and isoleucine, these amino acids have reactive functional side chains. It is very likely that the strong nucleophilicity of the thiol side chain^24^ leads to many of the observed MRPs produced in the ribose-cysteine model. Further, Munch *et al*.^25^ showed that, when the side chains were protected, the reactivity of N-terminal amino acids in dipeptides towards glucose and fructose addition is almost similar among the twenty proteinogenic amino acids. Only cysteine revealed slightly lower reactivity. However, when the side chains were unprotected cysteine and lysine showed by far highest reactivity^25^.

A simple and fast method to assess the progress of Maillard reactions is to measure the degree of browning. In the intermediate stage, non-enzymatic browning leads to chromophores showing good absorbance at 294 nm while 420 nm indicates reaction products of the final stage^26,27^. Here, after ten hours, the order in degree of browning found at 294 nm was: lysine >isoleucine >glycine >cysteine as shown in Fig. 1b. The same order was reported by Hwang *et al*. when they heated unbuffered amino acid – glucose mixtures for two hours at 130 °C^28^. For cysteine, we observed only a minor amount of browning over the entire reaction timescale. It has been reported in many studies that cysteine does not lead to extensively coloured compounds, but rather suppresses the formation of chromophores. The suppressing effect of cysteine has so far been mostly explained by the formation of relatively stable thiazolidines^29^ and its ability to effectively trap (di)carbonyls to form hemi- or thioacetals^30^. Although cysteine does not contribute to the characteristic browning in Maillard reactions, it is responsible for a huge diversity in reaction products as shown in our FT-ICR-MS data (Fig. 1a). Furthermore, cysteine is known to produce many meat-like aroma compounds including S-containing heterocyclic molecules which are often formed from cysteine degradation products such as H_2_S or cysteamine^31–33^. However, non-volatile Maillard intermediates, which are produced by cysteine, are largely unknown. With more than 400 MRPs observed in electrospray MS, we here report a large pool of chemical compounds which could act as non-volatile precursors in the formation of aromas. Moreover, although cysteine is often considered as relatively unreactive in the MR^29^ we could show that it is responsible for a variety of non-volatile intermediates, consequently having quite a remarkable reactivity.

Compared to the multitude of MRPs produced in the model systems, we observed only a few amino acid degradation products for lysine and cysteine (Fig. S2). No amino acid degradation products were observed for glycine and isoleucine in the covered mass range. Cysteine showed highest thermal instability resulting in 27 amino acid degradation products, which could be formed without the interaction with ribose. Some of those cysteine degradation products may also contribute to the total diversity in reaction products we observed in the ribose-cysteine MR. The number of sugar degradation products observed in the model systems is very similar for glycine, isoleucine and lysine (16 ± 2; Fig. S2). However, in the cysteine model system, the number of ribose decomposition products produced was considerably smaller than for the other amino acids. After ten hours, we found only nine ribose degradation products in the ribose-cysteine mixture. Thus, cysteine seems not only to suppress the browning in Maillard reactions but also has a suppressing effect on sugar decomposition. Sugar degradation products, such as furfural or dicarbonyls, significantly contribute to the degree of browning^4^. As a result, it can be assumed that suppression of sugar degradation products in cysteine-containing Maillard systems is a part of the suppression effect of cysteine on browning.

Degradation of the ARP and hence the composition and number of formed MRPs strongly depends, among many other factors, on the reaction pH^34,35^. In our reaction systems, pH values dropped with increasing reaction time. After ten hours, the pH decreased by approximately 2–3 pH units compared to unheated model systems. Acidification has a significant impact on the availability of amino groups and, to a lesser degree, an effect on the acyclic form of the sugar precursor^36^. The relative proportion of unprotonated amino groups, which is essential for nucleophilic reactions in the initial phase of the MR, increases with increasing pH. Lysine showed a strong increase in the formation of MRPs within the first four hours (Fig. 1a) which can be attributed to the availability of more amino groups in the early phase of the reaction.

### Compositional characteristics of MRPs

**Figure 2:**
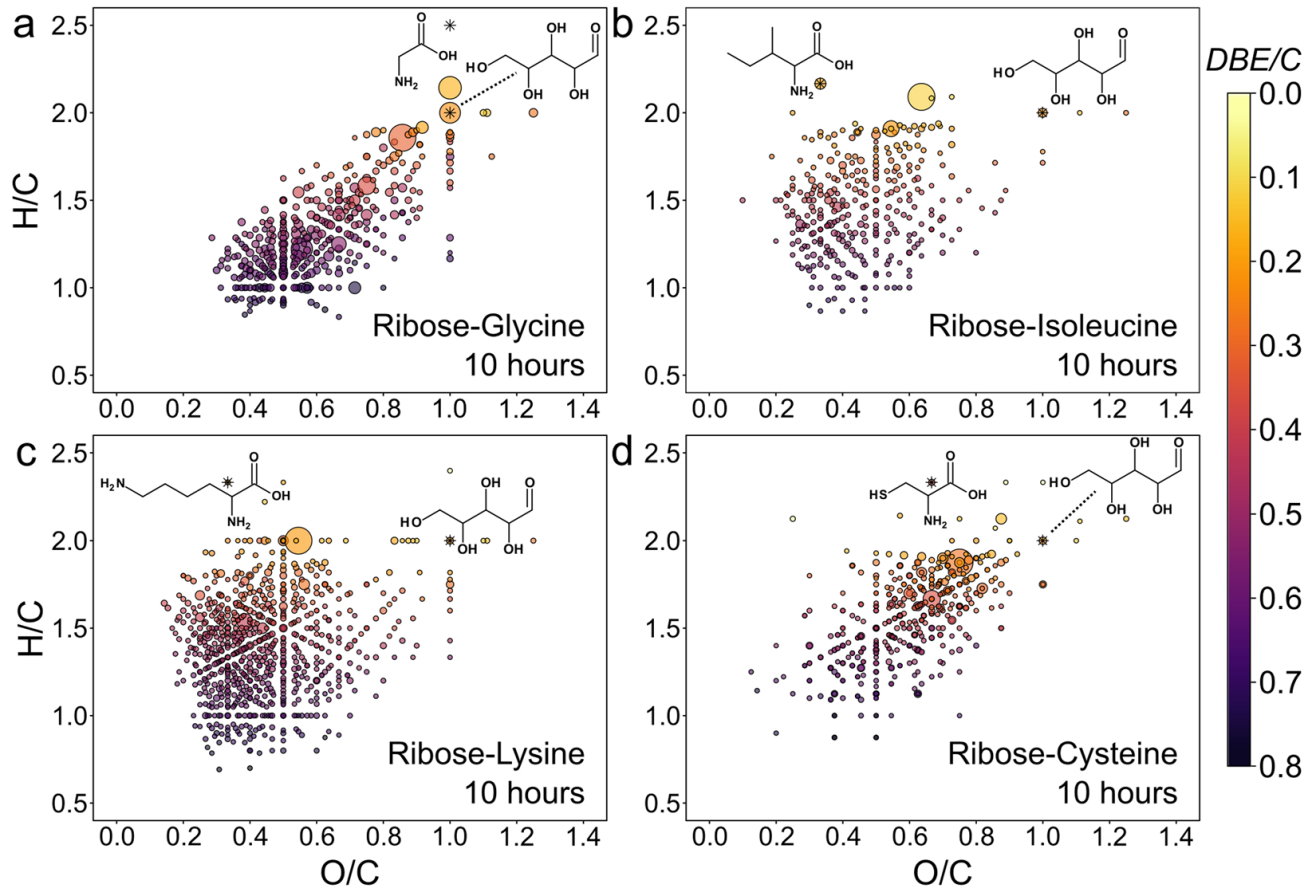
Compositional characterization of MRPs. (**a–d)** Van Krevelen diagrams (H/C vs. O/C atomic ratios) of four ribose-amino acid model systems heated for ten hours at 100 °C. Scaling of points is according to their relative peak intensity in the mass spectra. Colour gradient according to the degree in unsaturation (number of double bond equivalents per carbon atom (DBE/C)).

Among the many visualization tools used for high-resolution MS data, the van Krevelen diagram is a very valuable tool for the representation of hundreds or thousands of molecular compounds in a two-dimensional space^37^. The van Krevelen diagrams of the four investigated Maillard model systems show an extraordinary high molecular diversity in produced MRPs (Fig. 2). Primarily, the amino acid precursor is responsible for the individual characteristics of the detected MRPs. By comparison, when six different sugars were heated together with glycine for 24 h, the element compositions were very similar among the different sugars (Fig. S3). Different sugar precursors mainly revealed differences in the number of produced MRPs (ribose >arabinose >fructose ≈ xylose >galactose >glucose) but no substantial differences in their characteristic positions in the van Krevelen diagrams could be observed. Specifically, the sugar reactivity order can be attributed to a combination of factors, such as the proportion of free carbonyls in the sugar precursor^38,39^ and faster reaction rates of pentoses than hexoses^40^.

Interestingly, when comparing the molecular formulae of all four ribose amino acid model systems, there was no single molecular formula that was common to all four samples among the > 1000 MRPs identified. This demonstrates how difficult it is to find general Maillard reaction markers, which are independent of the amino acid and sugar precursors. It is worth noting, that ESI(−) analysis mainly targets oxygen-rich and polar MRPs of the initial and intermediate phase. In later stages of the MR an increasing similarity in chemical structures, *e.g*. after Strecker degradation can be expected. By comparison, molecular formulae retrieved for the different sugar-glycine model systems showed more similarity. After 24 h, 88%, 95%, 75%, and 78% of all observed MRPs in the arabinose- and xylose-, galactose-, and fructose-glycine mixtures were also found in the ribose-glycine mixture, respectively. Only the glucose-glycine model showed a smaller number of common formulae (45%) which is mainly because of the lower reactivity of glucose (Fig. S3). After 24 h we observed only a few initial MRPs formed in the glucose-glycine model. A longer reaction time might be required to initiate fragmentation of the sugar backbone, and may increase the molecular formulae intersect. Although chemical information obtained from direct-infusion FT-ICR-MS is restricted to accurate molecular formulae, the high proportion of common formulae between the different sugar systems indicates that different sugar precursors react to form similar or even identical MRPs. The many cleavage reactions might easily convert hexose-derived MRPs into smaller derivatives, which can also be formed directly from smaller carbohydrate precursors.

Compared to the glycine and cysteine mixtures, isoleucine and lysine showed a large number of MRPs with high H/C and low O/C ratios (aliphatic area, Fig. 2). The aliphatic parts of the side chains of isoleucine and lysine are mainly responsible for these specific molecular characteristics. However, the van Krevelen diagrams do not show a shift of all produced MRPs of the isoleucine and lysine models towards higher aliphaticity. We rather found a widespread distribution of MRPs over a large part of the van Krevelen space including also many unsaturated compounds (low H/C and O/C ratios, high DBE/C) similar to the MRPs produced in the ribose-glycine model system. Compounds with such low H/C ratios cannot contain original or intact lysine or isoleucine residues. More precisely, they must be produced either (i) by elimination of the amino acid side chain in one step of the reaction cascade, or (ii) by Maillard-type condensations of small amine intermediates with carbonyls. Indeed, 90% of the detected lysine-MRPs containing only one nitrogen atom showed H/C and O/C ratios not exceeding 1.5 and 0.6, respectively. Consequently, a part of the amino acid’s side chain must have been eliminated during the reaction. Eneaminols and amino ketones formed during the Strecker degradation (Fig. S4) could be an important class of compounds, which contain less aliphatic parts than the original amino acid and hence, would fit in the van Krevelen space of such low H/C and O/C ratios.

**Figure 3:**
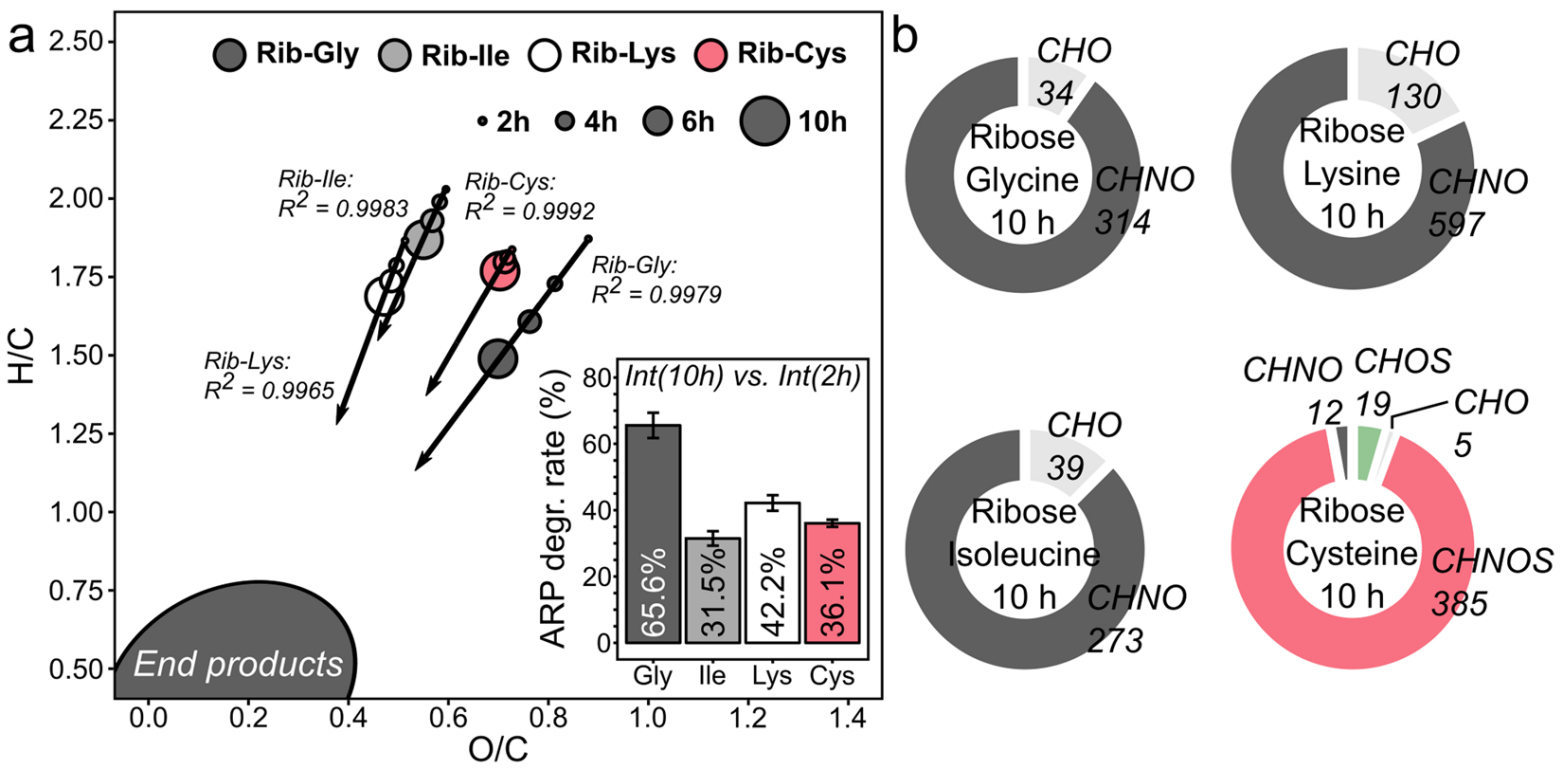
Changes in unsaturation over time and characteristics of compositional spaces. (**a)** Progression of H/C and O/C atomic ratios over time for ribose-glycine (dark grey), ribose-isoleucine (grey), ribose-lysine (white) and ribose-cysteine (red) MRPs. Points represent intensity weighted mean values of the H/C and O/C ratios, respectively. Linear regression lines visualize the direction of MRPs moving with increasing reaction time. (**b)** Absolute number of MRPs depicted for different chemical spaces.

Fundamentally, the contribution of MRPs of higher unsaturation increases with reaction time in all four model systems (Fig. 3a). However, glycine showed the highest tendency to form unsaturated compounds, followed by isoleucine and lysine. Cysteine showed only a very slow decrease in H/C and O/C ratios over time (Fig. 3a). This could be due to a much faster degradation of the ribose-glycine ARP. After ten hours, the relative intensities of the glycine-ARP decreased by 66% compared to the relative intensities observed after two hours. The degradation rates for the ARPs formed by the other amino acids were significantly lower (Fig. 3a). Extrapolation of the regression lines in Fig. 3a would lead to an intersection in an area of low H/C and O/C atomic ratios. There, highly unsaturated compounds, such as heterocycles like furfural, pyrazine, or pyrrole derivatives are found. Although these compounds are not detectable by ESI(−) the intersection represents an area where final products of the Maillard reaction (volatiles and polymer-type melanoidins) can be expected, including common MRPs, which can be produced by various amino acids. For example, Strecker degraded amino acids have been shown in the reaction with carbonyls to produce common pyrazine derivatives^41,42^.

We further divided and characterized the MRPs by their compositional spaces (Fig. 3b). Based on the amino acid precursor, glycine, isoleucine, and lysine can only produce MRPs containing carbon, hydrogen, oxygen, and nitrogen, thus compounds of the CHO and CHNO space. On the contrary, cysteine can additionally produce sulphur-containing MRPs (CHOS and CHNOS space). After ten hours, 89% of all detected MRPs (1268/1431) contained at least one nitrogen atom (CHNO or CHNOS space, Fig. 3b). Most of the N-containing MRPs in the cysteine Maillard reaction also contained sulphur. Only 12 sulphur-free but nitrogen containing MRPs could be identified contributing <1% to the total peak intensity. Thus, release of the cysteine side chain (*e.g*. by Strecker degradation) seems to have less impact than side chain elimination for example in the lysine Maillard reaction. We found 346 different molecular formulae in the ribose-lysine MR, accounting for 20% of the relative peak intensity, containing an odd number of nitrogen atoms (Fig. S5) indicating fragmentation of the amino acid in any step of the reaction progress. We also noticed significant differences in produced nitrogen-free (CHO) compounds among the four model systems (Fig. 3b). The number of different CHO-compounds was 34, 39, 130, and 5 for glycine, isoleucine, lysine, and cysteine, respectively. This again demonstrates the strong ability of cysteine to trap free carbonyl compounds.

### General Maillard reaction scheme

**Figure 4:**
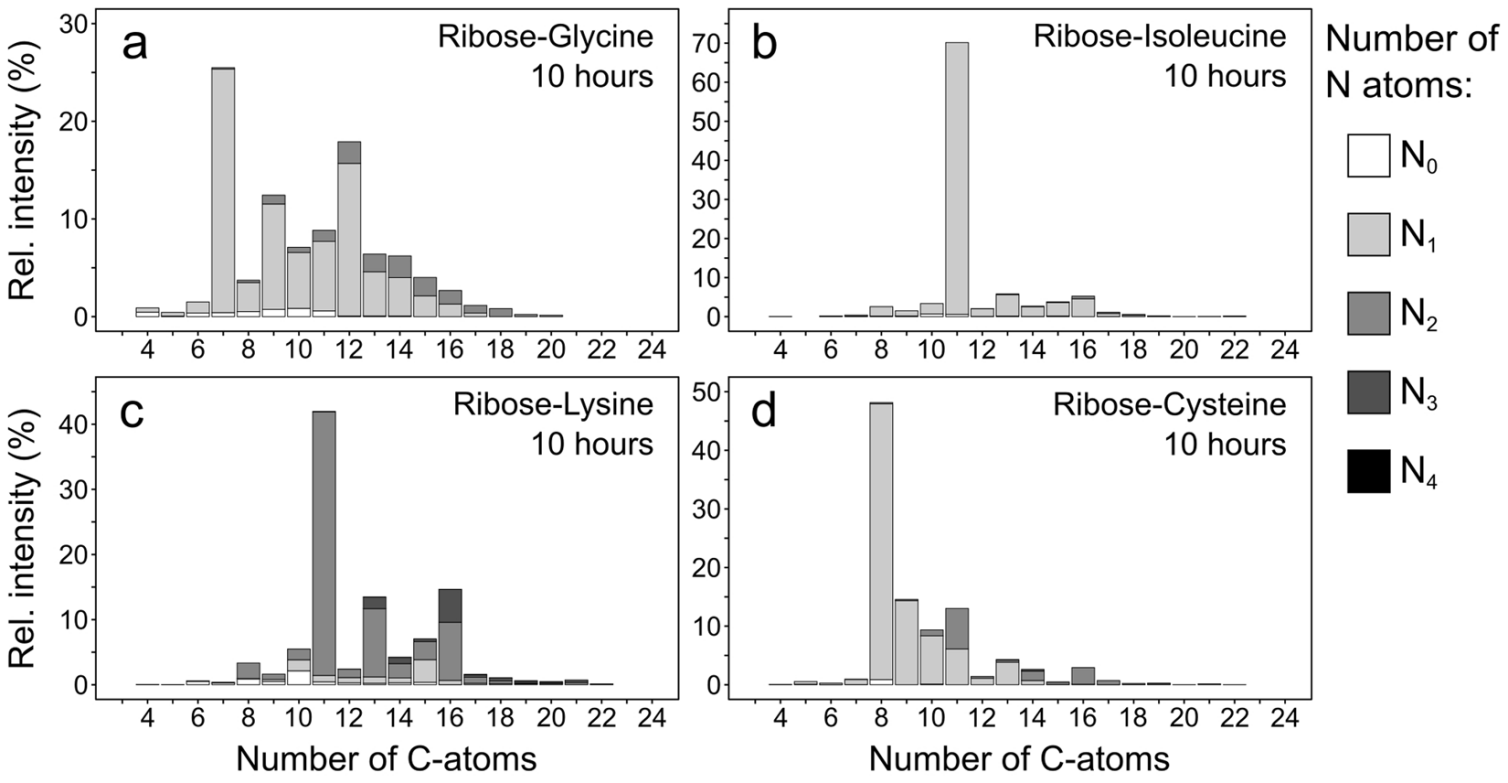
Number of carbon atoms in MRPs. Relative peak intensities classified by the number of carbon atoms of thermally synthesized (10 h, 100 °C) (**a)** ribose-glycine, (**b)** ribose-isoleucine, (**c)** ribose-lysine, and (**d)** ribose-cysteine MRPs.

Next, we analysed the MRPs based on their carbon backbone (Fig. 4). We found for all model systems highest intensities for the carbon chain lengths which result from the reaction of one molecule of ribose with one molecule of amino acid (glycine: C_7_, isoleucine/lysine: C_11_, and cysteine: C_8_) indicating that the formation and direct degradation pathway of the ARP contributes most to the formation of MRPs. Furthermore, much higher intensity contributions, divergent from an assumed normal distribution, were found for several carbon chain lengths: the reaction of two molecules of ribose with one amino acid molecule (glycine: C_12_, isoleucine/lysine: C_16_, and cysteine: C_13_) accounted for 16% to the total ribose-glycine MR intensity. Additionally, we found higher intensities for ribose + amino acid + C_2_ (or 2 ribose + amino acid – C_3_) in the glycine, isoleucine, and lysine models (Fig. 4a–c) as well as for ribose + amino acid + C_3_ (or 2 ribose + amino acid – C_2_) in the ribose-cysteine model (Fig. 4d). The latter observation revealed that approx. half of the produced MRPs with C_11_ contained two nitrogen atoms. In the other model systems (glycine, isoleucine, and lysine), MRPs resulting from the reaction of one molecule ribose with two molecules of amino acid did not contribute significantly to the total intensity. We assume that the C_11_ reaction products containing two nitrogen atoms found in the cysteine model arise from the reaction of earlier formed cystine and ribose or the oxidation of C_8_-MRPs with another molecule of cysteine under formation of a disulphide link. This also explains the higher intensity contribution found for C_16_ in the ribose-cysteine samples.

**Figure 5:**
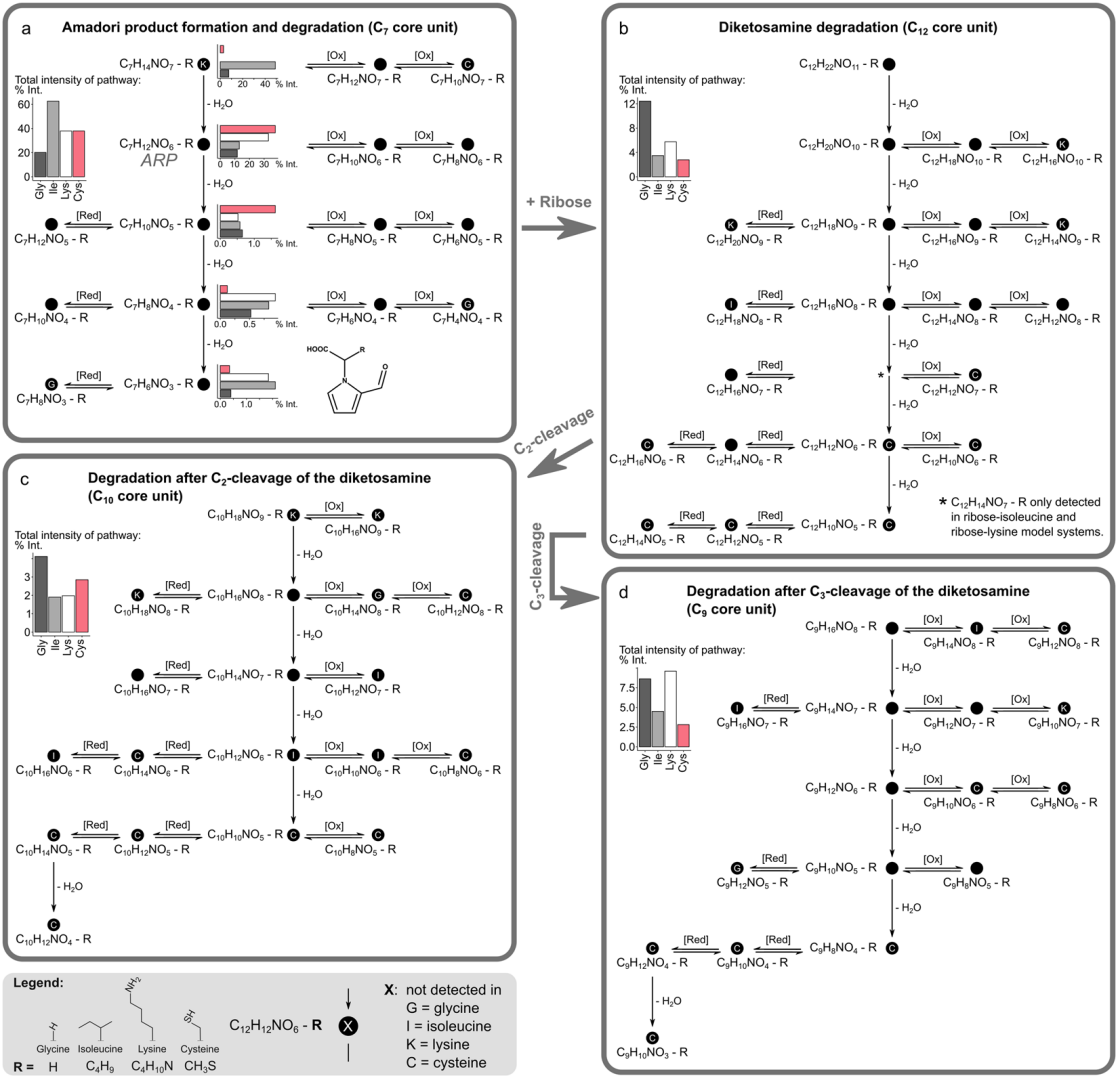
General Maillard reaction product formation and degradation pathways. Maillard reaction products with identical element core compositions detected (S/N ratio ≥ 8) in at least three of the four investigated Maillard model systems revealed identical reaction behaviour following a well-defined series of dehydration and redox reactions. The scheme could be sub-divided into four pathways of similar assembly. (**a)** ARP formation and degradation, (**b)** Diketosamine degradation, (**c**,**d)** C_2_- and C_3_-cleavage. Molecular formulae shown have the same core composition but differ in the amino acid residues (−R). Embedded bar charts illustrate the relative peak intensity contributions after ten hours.

Most of the MRPs bearing the discussed carbon chain lengths turned out to follow identical reactivity patterns and have compositional similarities. All MRPs, which were found in at least three out of the four model systems, heated for ten hours, followed identical reactivity rules and had the same core composition, are summarized in Fig. 5. We found more than 70 different molecular compositions (molecular formulae) which only differ in the amino acid side chain. In sum, they explained 45%, 73%, 55%, and 46% of the total peak intensity found for the ribose-glycine, -isoleucine, -lysine, and -cysteine MRPs, respectively. The candidates can be further subdivided into four pathways of similar assembly. First, the reaction between one molecule of ribose and an amino acid molecule builds the Amadori product formation and degradation scheme (Fig. 5a). The ARP then may react with a second molecule of ribose leading to highly reactive diketosamines (Fig. 5b). Diketosamines, such as n,n-bis(1-deoxy-d-erythro-2-pentulos-1-yl)-glycine, are assumed to decompose much faster than the initial ARP^43^ and may thereby subsequently undergo C_2_- and C_3_-cleavage reactions (Fig. 5c,d).

Each of the reaction schemes in Fig. 5 comprises of a series of dehydration reactions. Additionally, several redox reactions may arise from the dehydrated intermediates. Carbonyls produced during dehydration can rapidly interconvert to ene-aminol and ene-diol structures which are known to undergo reversible redox behavior^44^. As for most of the intermediates in the dehydration series, both reduced and oxidized compounds were found; it is very likely, that behind each of the 73 “general” MR compounds several different chemical isomers are conceivable. Although the relative intensities of the oxidized and reduced MRPs were always lower than those depicted as dehydration products, it is possible that oxidation products especially, act as important intermediates in the dehydration process. Oxidation of hydroxyl groups could lead to carbonyls acting as targets for intramolecular nucleophilic additions with subsequent dehydration, finally, increasing the dehydration reaction rates.

### ARP formation and degradation scheme

With exception of the isoleucine-ARP, all ARPs were observed as base peaks in the respective mass spectra. With increasing reaction time, we observed up to three ARP-dehydration products (Fig. 5a). Relative intensities of the ARP-dehydration products decreased with increasing dehydration progress. However, intensities of the ARP-3⋅H_2_O were again higher than for the ARP-2⋅H_2_O indicating a more stable molecule such as the substituted 2-formyl-1-pyrrole-1-acetate as shown in Fig. 5a. 2-Formyl-1-pyrrole-1-acetate derivatives could be isolated when xylose was heated with glycine^45^ and isoleucine^46^. Higher relative peak intensities (>30%) were observed for the cysteine- and lysine-ARPs as compared to the glycine- and isoleucine-ARPs. This shows that the initial condensation rates of cysteine and lysine are, due to the reactive side chain, higher than for glycine and isoleucine which is in agreement with the findings of Munch *et al*.^25^.

### Diketosamine degradation scheme

While ribose-glycine MRPs accounted for only 20% of the total intensity in the Amadori degradation pathway MRPs in the diketosamine degradation scheme were found to explain up to three times more of the total intensity than the other amino acids (Fig. 5b). This could be due to steric hindrance caused by the bulky isoleucine residue or intramolecular cyclization through the nucleophilic lysine and cysteine residues hindering further carbonyl condensation. The starting point of the diketosamine degradation pathway results from the addition of the ARP (C_7_H_12_NO_6_-R) to a second ribose molecule. In total, we found six possible dehydration products which could arise from this initial addition reaction. However, the structural variety of molecules is expected to be even higher than in the Amadori degradation pathway. Several combining possibilities exist which could lead to stable molecules involved in this pathway. It is worth noting, that there is no evidence for a complete series of successive dehydration events actually taking place. The pathways must rather be understood as a conceptual summary of dominating and regular patterns we observed between the identified molecular formulae. For instance, it is also conceivable that the last dehydration product in Fig. 5b (C_12_H_10_NO_5_-R) results from a condensation reaction between the substituted 2-formyl-1-pyrrole-1-acetate (C_7_H_6_NO_3_-R in Fig. 5a) and norfuraneol as shown by Ledl and Severin^45^.

### C2- and C3- cleavage schemes

The C_2_- and C_3_-cleavage pathways (Fig. 5c,d) arise most likely from α- and β-dicarbonyl cleavage of intermediates from the diketosamine degradation pathway (Fig. 5b). Different cleavage mechanisms can be taken into account to explain the resulting molecular compositions of the pathways in Fig. 5c,d, such as retroaldolization^35^ or many other putative or proven (di)-carbonyl cleavage mechanisms. For example, in a C_2_-cleavage reaction, C_2_H_4_O_2_, acetic or glycolic acid could be possible by-products. For glycine, isoleucine, and lysine C_3_-cleavage reactions seem to be preferred relative to C_2_-cleavage, which is exemplified by the higher peak intensities. By comparison, cysteine derived MRPs showed similar or slightly higher intensities explained by the C_2_-pathway. Interestingly, late dehydration products and the linked oxidized and reduced MRPs in Fig. 5b–d could not be detected in the ribose-cysteine samples indicating a different reactivity of cysteine MRPs in the final steps of the chemical pathways. Cysteine is known to react somewhat differently compared to other amino acids. It can be assumed that the strong ability of the cysteine side chain to undergo redox reactions is in competition with redox activities on the carbohydrate backbone. This can explain lower intensities of ribose-cysteine MRPs and the different reactivity particularly in steps involving oxidation or reducing steps.

## Conclusion

In the present fundamental study, we showed that non-volatile initial and intermediate Maillard reaction products can be comprehensively analysed using direct-infusion FT-ICR-MS, hence, bypassing the restricted selectivity of LC-based methods and finally delivering accurate molecular formulae for the entire reaction products. Here, we mainly compared four ribose-amino acid Maillard model systems using amino acids with very different side chains (glycine, isoleucine, lysine, and cysteine). Depending on the amino acid precursor, we observed wide disparity in the absolute number and compositional characteristics of produced MRPs. The order of reactivity we found after heating the model systems for ten hours at 100 °C was: ribose-lysine >-cysteine >-isoleucine ≈ -glycine. While the amino acid precursors are responsible for the molecular characteristics of MRPs, sugar precursors drive the reaction rates. Even though cysteine is often considered as relatively unreactive in the Maillard reaction, surprisingly, it produced more than 400 distinct MRPs. Many of those MRPs are apparently produced through condensation reactions between the nucleophilic thiol residue and carbonyls as well as oxidative formation of disulphide links resulting in reaction products, which, in this way, cannot be produced by any other proteinogenic amino acid. Further studies in a more complex environment, such as mixtures of several amino acids or protein hydrolysates, may help to better understand the particular role of cysteine derived MRPs and the ability of thiols to effectively suppress browning.

Although we could not detect a single molecular formula, apart from ribose degradation products, which was found in all four model systems, more than 70 MRPs were identified that followed similar reactivity behaviour. These “general” MRPs have identical element core compositions differing only in the amino acid side chain and could be further classified into Amadori product degradation, diketosamine degradation and two different carbonyl cleavage pathways. To date, little is known about the role of diketosamines in the MR. However, based on our findings, we can conclude that the early formation and subsequent degradation of diketosamines contribute to a large extend to the overall MRPs. Mechanistic studies should be performed in order to fully understand exact reaction routes and rates between the MRPs in the proposed degradation pathways. Furthermore, it is yet unclear whether other reaction conditions (*e.g*. pH, temperature, water content), which are relevant under physiological conditions or in food samples, would lead to similar ARP degradation behaviour. Hence, further studies are required, which specifically investigate the formation of MRPs under the reaction conditions of interest.

## Methods

Mixtures of ribose and amino acids (0.1 mol L^−1^ respectively) were prepared in Milli-Q purified water (Millipore, Germany). 1 mL of each mixture was heated in 2 mL glass vials sealed with temperature and pressure resistant crimp caps to exclude additional air/gas exchange. For the identification of ribose and amino acid degradation products, blank samples containing 0.1 mol L^−1^ ribose or amino acid were prepared. All samples were diluted 1:500 (v/v) with methanol (LC-MS grade, Fluka, Germany) and analysed by direct-infusion FT-ICR-MS as recently described^14^. All Maillard model systems were prepared and analysed in triplicate. Further details are given in SI Materials and Methods.

## Electronic supplementary material

Supporting Information

## Data Availability

The datasets generated or analysed during this study are included in this published article and its Supplementary Information files.
