# Supplementary material for: Insights into the Chemistry of Non-Enzymatic Browning Reactions in Different Ribose-Amino Acid Model Systems
Source: Sci Rep. 2018 Nov 15;8:16879. doi: 10.1038/s41598-018-34335-5 (PMC6237888; doi:10.1038/s41598-018-34335-5)
Supplement: Supplementary file 1 — Supporting Information [file 41598_2018_34335_MOESM1_ESM.pdf]

Insights into the Chemistry of Non-Enzymatic Browning Reactions in Different Ribose-Amino Acid Model Systems

Supporting Information

Daniel Hemmler[1,2]*, Chloé Roullier-Gall[1,2], James W. Marshall[3], Michael Rychlik[1], Andrew J. Taylor[3], Philippe Schmitt-Kopplin[1,2]*

[1]Comprehensive Foodomics Platform, Analytical Food Chemistry, Technical University Munich, Alte Akademie 10, 85354 Freising, Germany.

[2]Research Unit Analytical BioGeoChemistry (BGC), Helmholtz Zentrum München, Ingolstädter Landstrasse 1, 85764 Neuherberg, Germany.

[3]The Waltham Centre for Pet Nutrition, Mars Petcare UK, Waltham-on-the-Wolds, Leicestershire, LE14 4RT, United Kingdom.

Table of contents

Materials and methods S2

Direct infusion FT-ICR-MS spectra S3

Classification of reaction products S4

Consideration of different sugar precursors in the glycine-Maillard reaction S5

Formation of eneaminols and amino ketones during the Strecker degradation S6

Compositional Characterization of MRPs S7

"General" Maillard reaction products formation and degradation pathways S8

References (Supporting Information) S12

Materials and methods

Chemicals. D-(-)-Ribose (Rib, 98%), L-lysine (Lys, ≥ 99%), L-cysteine (Cys, ≥ 97%), and L-isoleucine (Ile, 99%) were purchased from Sigma-Aldrich (Steinheim, Germany). Glycine (Gly, ≥ 98.5%) was obtained from Merck (Darmstadt, Germany).

Model systems. Mixtures of ribose and amino acids (0.1 mol L^{-1} respectively) were prepared in Milli-Q purified water (Millipore, Germany) immediately prior to thermal treatment. Ribose-amino acid mixtures were heated in closed glass vials for two, four, six and ten hours in boiling water (100 °C). For the identification of ribose and amino acid degradation products, blank samples containing 0.1 mol L^{-1} ribose or amino acid were prepared. All Maillard model systems were prepared and analysed in triplicate.

Direct infusion FT-ICR mass spectrometry. Direct-infusion FT-ICR mass spectra were acquired with a 12 Tesla Bruker Solarix FT-ICR mass spectrometer (Bruker Daltonics, Bremen, Germany). The MS was first calibrated by means of arginine ion clusters (57 nmol mL^{-1} in methanol). Next, raw spectra were further internally calibrated using a reference list including known Maillard reaction markers and ubiquitous fatty acids to achieve best possible mass accuracy and precision among the samples. Raw spectra were post-processed by Compass DataAnalysis 4.2 (Bruker Daltonics, Bremen, Germany) and peaks with a signal-to-noise ratio (S/N) of at least 8 were exported to mass lists. All exported features were aligned in a matrix containing averaged *m/z*-values (maximum peak alignment window width: ± 1 ppm) and corresponding peak intensities of all analysed samples[1]. Only *m/z* features of monoisotopic candidates and features with feasible mass defect were retained in the matrix. Identification of heavy isotope candidates was performed as described elsewhere[2]. Remaining *m/z*-values were assigned to their unambiguous molecular formulae as recently described[3]. Chloride adducts $[M+Cl]^-$ were only retained in the final data matrix when no corresponding $[M-H]^-$ ion was found. In general, chloride adduct formation played only a minor role. In the ribose-lysine model systems 26 and in the ribose-cysteine model systems one $[M+Cl]^-$ adducts could be detected which were not recorded as $[M-H]^-$. No such unique chloride adducts were found for ribose-glycine and ribose-isoleucine MRPs.

UV-absorbance. Samples were diluted 1:100 (v/v) with Milli-Q purified water (Millipore, Germany). Immediately after dilution, the absorbance at 294 nm was measured using a µQuant Spectrophotometer (Bio-Tek Instruments, USA).

Data analysis. All further data processing was done in Microsoft Excel 2010 and R Statistical Language (version 3.4.1)[4]. Only those molecular features, which were detected in all three replicates (S/N ≥ 8) of one sample group, were considered for further data analysis and interpretation. The number of double-bond equivalents (sum of rings and double bonds in a molecule) per carbon atom (DBE/C) can be calculated according to Eqn. 1 from the number of atoms (n_i) and the valence (v_i) of each element i.

$$\mathrm{DBE/C} = \frac{1 + \frac{1}{2}\sum n_i(v_i - 2)}{n_C} \qquad (1)$$

Direct infusion FT-ICR-MS spectra

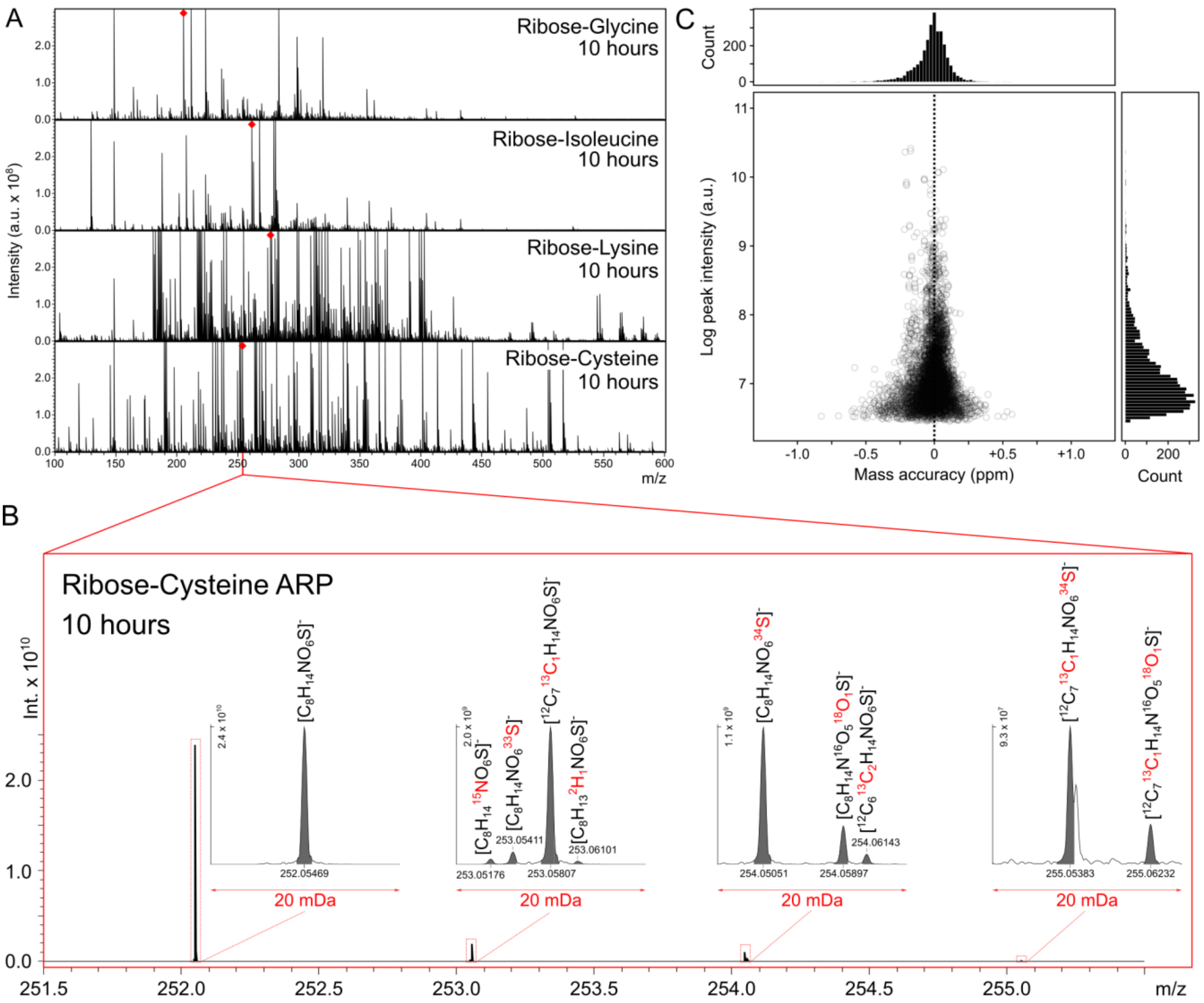

Fig. S1 Direct infusion FT-ICR-MS spectra. **a** Raw spectra of four different ribose-amino acid model systems heated for ten hours (100 °C). Red diamonds indicate the position of the Amadori rearrangement products in the mass spectra. **b** Isotopic fine structure validation of the ribose-cysteine Amadori product ion ($C_8H_{14}NO_6S^-$) by means of the exact masses and relative abundances of nine isotope signals. **c** Mass accuracy and peak intensity of 1493 monoisotopic peaks assigned to their molecular formulae.

Classification of reaction products

Molecular formulae were classified according to Yaylayan into three different reaction product pools[5]: (i) Maillard reaction products (MRPs), (ii) thermal induced carbohydrate degradation products, and (iii) amino acid degradation products. Ion signals, which were found exclusively in all three replicates of the model systems but not in the blank samples (ribose and amino acids heated alone) were classified as MRPs. Features also found in the ribose blank sample were classified as carbohydrate decomposition products. Those features, which were found in the model systems and the amino acid blank sample, were classified as amino acid decomposition products.

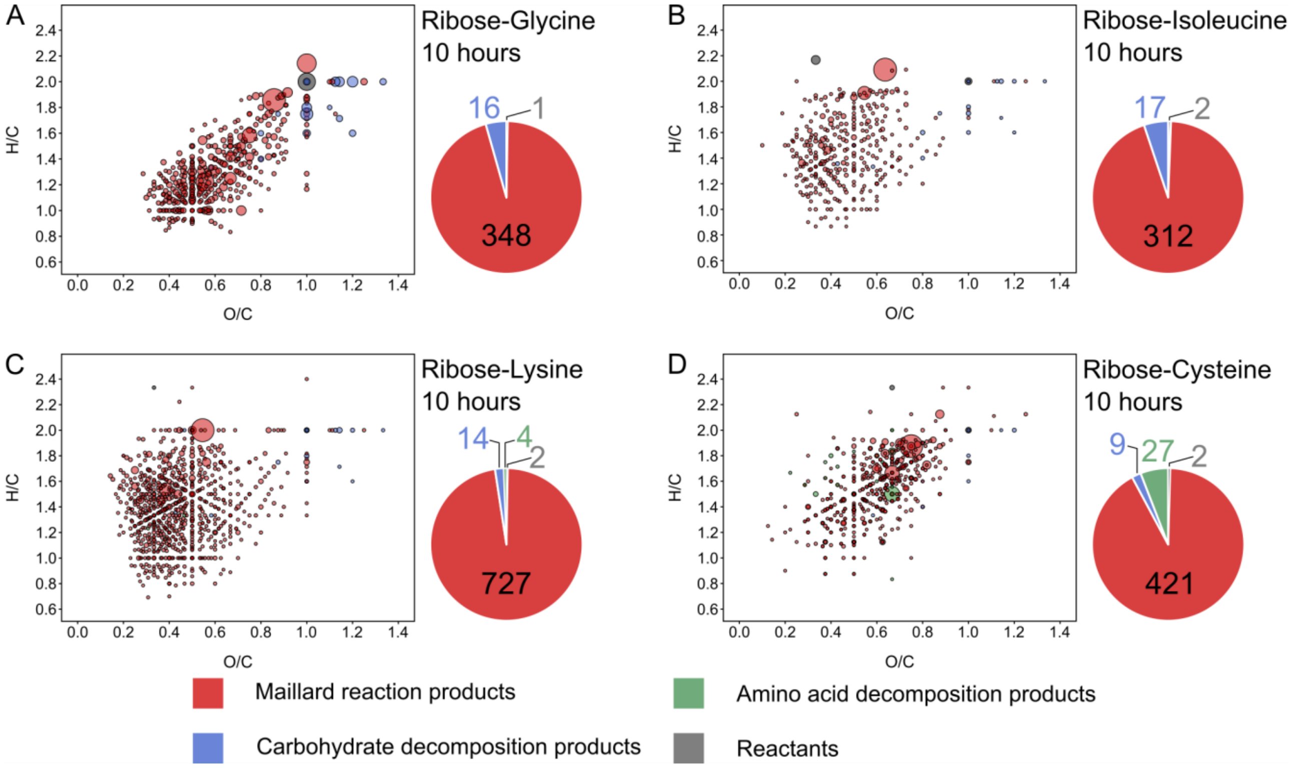

Fig. S2 Classification of detected reaction products as Maillard reaction products (MRPs), carbohydrate and amino acid degradation products.

Consideration of different sugar precursors in the glycine Maillard reaction

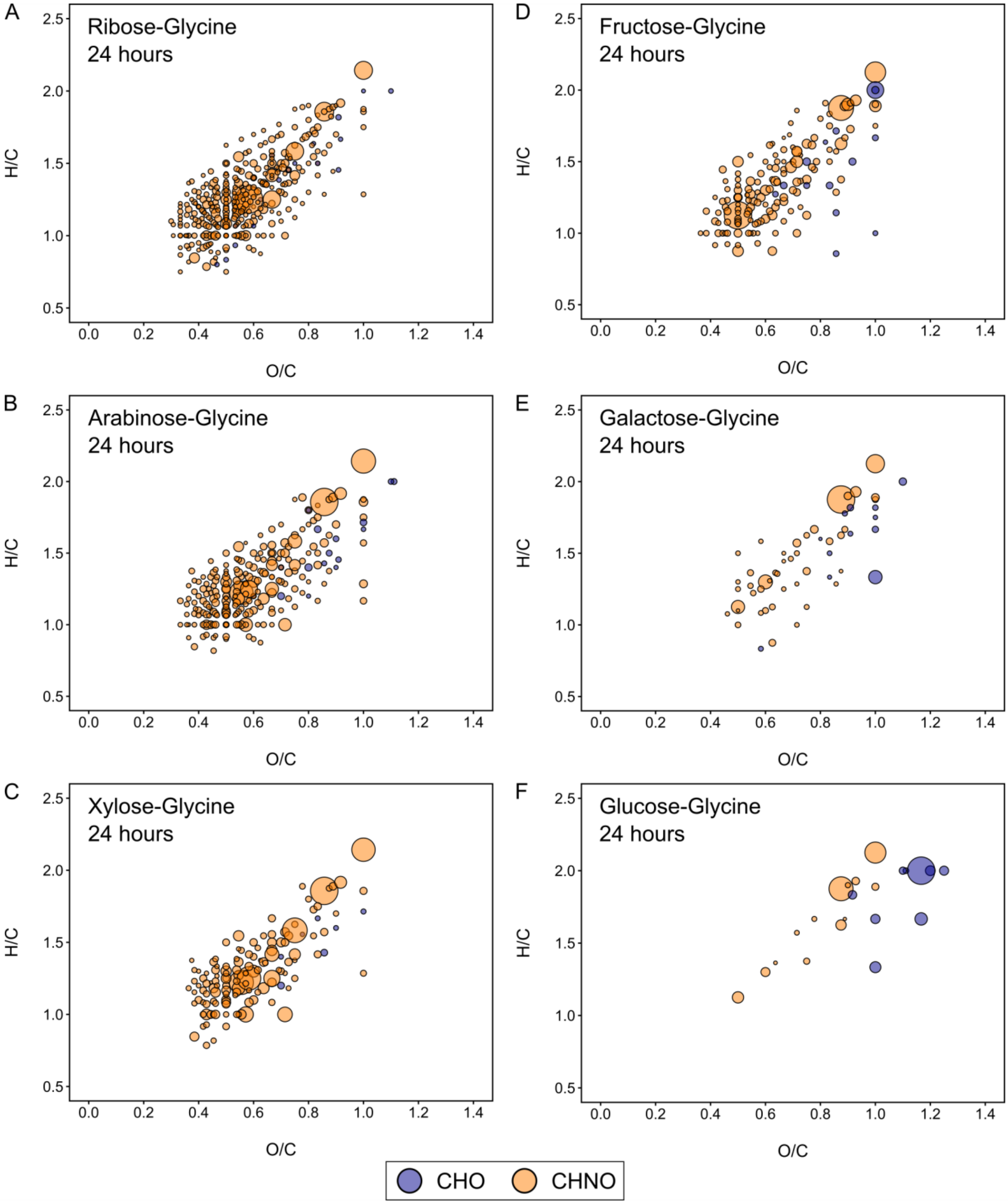

Fig. S3 Van Krevelen diagrams of MRPs found in three different pentose-glycine **a-c** and three hexose-glycine **d-f** Maillard reaction model systems heated for 24 h at 100 °C.

Formation of eneaminols and amino ketones during the Strecker degradation

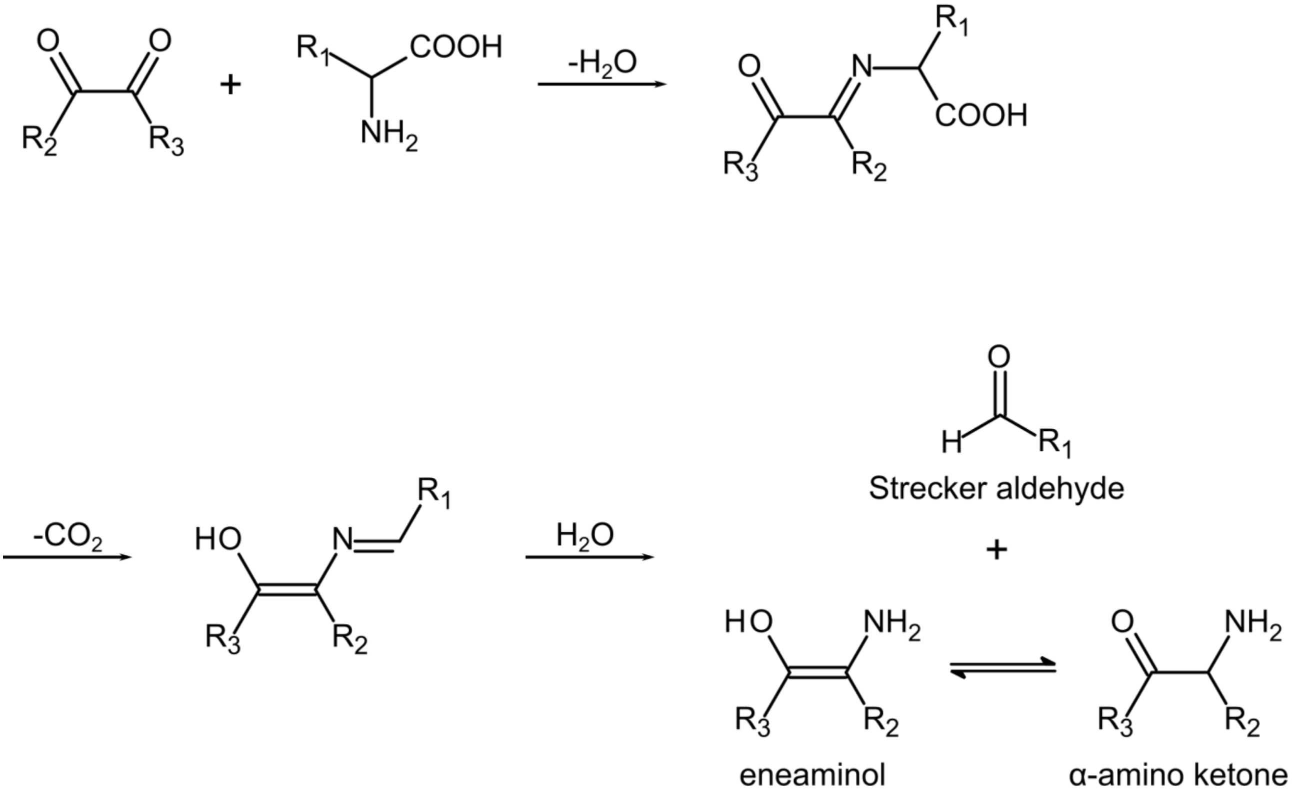

Fig. S4 Strecker degradation in the Maillard reaction. Eneaminols and amino ketones formed in the Strecker degradation of amino acids by dicarbonyls could be a class of compounds with low H/C and O/C ratios as observed in the lysine and isoleucine Maillard reaction.

Compositional Characterization of MRPs

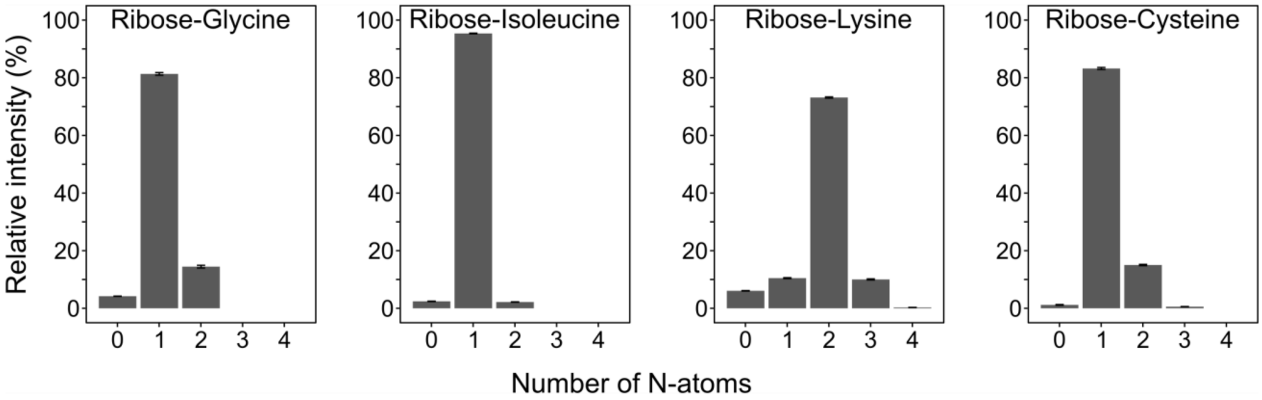

Fig. S5 Relative peak intensities explained by MRPs classified by the number of nitrogen atoms in the molecular formulae of model systems heated for ten hours (100 °C).

“General” Maillard reaction products formation and degradation pathways

Table S1 Ribose-glycine derived Maillard reaction products which appear in the "general" Maillard reaction products formation and degradation pathways shown in Fig. 5; (n.d.) not detected or S/N < 8.

				Relative peak intensity (mean ± sd, n = 3)			
**General Formula**	**Ribose-glycine**	***m/z* (avg.)**	**error (ppm)**	**2h**	**4h**	**6h**	**10h**
C7H12NO6 - R	C7H13NO6	206.067007	-0.029	0.4091 ± 0.0098	0.3096 ± 0.0159	0.1929 ± 0.0144	0.1168 ± 0.0103
C7H14NO7 - R	C7H15NO7	224.077577	-0.003	0.2573 ± 0.0105	0.1622 ± 0.0043	0.1075 ± 0.0036	0.0758 ± 0.0049
C7H6NO4 - R	C7H7NO4	168.030239	0.036	n.d.	0.0083 ± 0.0005	0.0101 ± 0.0011	0.0107 ± 0.0004
C7H6NO3 - R	C7H7NO3	152.035318	-0.003	n.d.	n.d.	0.0034 ± 0.0002	0.0040 ± 0.0003
C7H10NO5 - R	C7H11NO5	188.056450	0.008	0.0110 ± 0.0000	0.0106 ± 0.0008	0.0095 ± 0.0009	0.0065 ± 0.0000
C7H6NO5 - R	C7H7NO5	184.025158	0.056	0.0054 ± 0.0007	0.0100 ± 0.0009	0.0140 ± 0.0018	0.0138 ± 0.0010
C7H8NO4 - R	C7H9NO4	170.045892	0.053	n.d.	0.0060 ± 0.0004	0.0048 ± 0.0003	0.0052 ± 0.0001
C7H10NO6 - R	C7H11NO6	204.051363	0.001	0.0055 ± 0.0010	0.0078 ± 0.0011	0.0080 ± 0.0012	0.0047 ± 0.0001
C7H12NO7 - R	C7H13NO7	222.061924	-0.019	n.d.	n.d.	0.0034 ± 0.0008	0.0028 ± 0.0002
C7H8NO5 - R	C7H9NO5	186.040806	0.040	n.d.	0.0044 ± 0.0004	0.0046 ± 0.0002	0.0031 ± 0.0002
C7H10NO4 - R	C7H11NO4	172.061541	0.048	n.d.	n.d.	n.d.	0.0014 ± 0.0001
C7H8NO6 - R	C7H9NO6	202.035705	-0.040	n.d.	n.d.	0.0020 ± 0.0002	0.0013 ± 0.0001
C7H10NO7 - R	C7H11NO7	220.046274	-0.018	n.d.	n.d.	n.d.	0.0009 ± 0.0001
C7H12NO5 - R	C7H13NO5	190.072082	-0.084	n.d.	n.d.	n.d.	0.0006 ± 0.0000
C7H4NO4 - R	C7H5NO4	n.d.	n.d.	n.d.	n.d.	n.d.	n.d.
C7H8NO3 - R	C7H9NO3	n.d.	n.d.	n.d.	n.d.	n.d.	n.d.
C12H18NO9 - R	C12H19NO9	320.098692	-0.052	0.0287 ± 0.0015	0.0442 ± 0.0009	0.0431 ± 0.0033	0.0411 ± 0.0008
C12H14NO8 - R	C12H15NO8	300.072498	0.018	n.d.	0.0157 ± 0.0008	0.0240 ± 0.0018	0.0247 ± 0.0020
C12H16NO8 - R	C12H17NO8	302.088135	-0.025	0.0127 ± 0.0018	0.0203 ± 0.0012	0.0180 ± 0.0006	0.0154 ± 0.0004
C12H22NO11 - R	C12H23NO11	356.119789	-0.138	0.0543 ± 0.0035	0.0372 ± 0.0023	0.0213 ± 0.0020	0.0139 ± 0.0015
C12H16NO9 - R	C12H17NO9	318.083061	0.009	n.d.	0.0067 ± 0.0009	0.0087 ± 0.0008	0.0100 ± 0.0001
C12H20NO10 - R	C12H21NO10	338.109237	-0.107	0.0248 ± 0.0157	0.0168 ± 0.0201	0.0057 ± 0.0013	0.0062 ± 0.0046
C12H14NO6 - R	C12H15NO6	268.082673	0.037	n.d.	0.0049 ± 0.0007	0.0051 ± 0.0002	0.0054 ± 0.0003
C12H12NO6 - R	C12H13NO6	266.067022	0.033	n.d.	n.d.	0.0040 ± 0.0002	0.0054 ± 0.0004
C12H12NO7 - R	C12H13NO7	282.061931	0.009	n.d.	n.d.	0.0048 ± 0.0006	0.0048 ± 0.0002
C12H16NO10 - R	C12H17NO10	334.077944	-0.086	n.d.	n.d.	0.0039 ± 0.0010	0.0041 ± 0.0001
C12H16NO7 - R	C12H17NO7	286.093228	-0.002	n.d.	n.d.	0.0032 ± 0.0007	0.0035 ± 0.0001
C12H12NO8 - R	C12H13NO8	298.056833	-0.033	n.d.	n.d.	0.0035 ± 0.0006	0.0027 ± 0.0001
C12H14NO5 - R	C12H15NO5	252.087752	0.016	n.d.	n.d.	0.0024 ± 0.0003	0.0026 ± 0.0001
C12H20NO9 - R	C12H21NO9	322.114365	0.022	n.d.	n.d.	n.d.	0.0019 ± 0.0000
C12H18NO10 - R	C12H19NO10	336.093557	-0.196	n.d.	n.d.	0.0025 ± 0.0003	0.0018 ± 0.0001
C12H12NO5 - R	C12H13NO5	250.072107	0.037	n.d.	n.d.	n.d.	0.0018 ± 0.0001
C12H14NO9 - R	C12H15NO9	316.067426	0.057	n.d.	n.d.	n.d.	0.0017 ± 0.0001
C12H16NO6 - R	C12H17NO6	270.098322	0.031	n.d.	n.d.	n.d.	0.0016 ± 0.0000
C12H10NO5 - R	C12H11NO5	248.056447	-0.005	n.d.	n.d.	n.d.	0.0015 ± 0.0001
C12H10NO6 - R	C12H11NO6	264.051353	-0.038	n.d.	n.d.	n.d.	0.0015 ± 0.0001
C12H18NO8 - R	C12H19NO8	304.103791	-0.006	n.d.	n.d.	n.d.	0.0012 ± 0.0003
C10H12NO6 - R	C10H13NO6	242.067014	0.003	0.0077 ± 0.0011	0.0144 ± 0.0004	0.0144 ± 0.0006	0.0103 ± 0.0003
C10H12NO5 - R	C10H13NO5	226.072077	-0.094	n.d.	0.0126 ± 0.0007	0.0142 ± 0.0005	0.0099 ± 0.0002
C10H10NO5 - R	C10H11NO5	224.056419	-0.129	n.d.	0.0048 ± 0.0006	0.0049 ± 0.0004	0.0042 ± 0.0003
C10H14NO7 - R	C10H15NO7	260.077585	0.026	n.d.	0.0045 ± 0.0002	0.0043 ± 0.0001	0.0032 ± 0.0000
C10H10NO6 - R	C10H11NO6	240.051366	0.013	n.d.	n.d.	0.0037 ± 0.0004	0.0030 ± 0.0001
C10H16NO9 - R	C10H17NO9	294.083063	0.018	n.d.	n.d.	0.0031 ± 0.0005	0.0027 ± 0.0001
C10H12NO7 - R	C10H13NO7	258.061938	0.038	n.d.	n.d.	0.0032 ± 0.0006	0.0026 ± 0.0002
C10H14NO5 - R	C10H15NO5	228.087729	-0.083	n.d.	n.d.	0.0025 ± 0.0005	0.0025 ± 0.0002
C10H14NO6 - R	C10H15NO6	244.082659	-0.018	n.d.	n.d.	0.0028 ± 0.0003	0.0024 ± 0.0001
C10H16NO8 - R	C10H17NO8	278.088150	0.027	0.0056 ± 0.0017	n.d.	0.0028 ± 0.0008	0.0017 ± 0.0003
C10H12NO4 - R	C10H13NO4	210.077175	-0.037	n.d.	n.d.	n.d.	0.0016 ± 0.0001
C10H16NO7 - R	C10H17NO7	262.093232	0.016	n.d.	n.d.	n.d.	0.0011 ± 0.0001
C10H18NO9 - R	C10H19NO9	296.098694	-0.046	n.d.	n.d.	n.d.	0.0010 ± 0.0001
C10H8NO5 - R	C10H9NO5	222.040775	-0.102	n.d.	n.d.	n.d.	0.0010 ± 0.0000
C10H8NO6 - R	C10H9NO6	238.035718	0.022	n.d.	n.d.	n.d.	0.0009 ± 0.0001
C10H18NO8 - R	C10H19NO8	280.103780	-0.046	n.d.	n.d.	n.d.	0.0009 ± 0.0001
C10H12NO8 - R	C10H13NO8	274.056877	0.123	n.d.	n.d.	n.d.	0.0009 ± 0.0001
C10H16NO6 - R	C10H17NO6	246.098312	-0.004	n.d.	n.d.	n.d.	0.0008 ± 0.0001
C10H14NO8 - R	C10H15NO8	n.d.	n.d.	n.d.	n.d.	n.d.	n.d.
C9H16NO8 - R	C9H17NO8	266.088145	0.006	n.d.	0.0081 ± 0.0009	0.0060 ± 0.0006	0.0049 ± 0.0004
C9H12NO8 - R	C9H13NO8	262.056857	0.052	n.d.	n.d.	n.d.	0.0007 ± 0.0001
C9H14NO8 - R	C9H15NO8	264.072450	-0.163	n.d.	n.d.	n.d.	0.0007 ± 0.0001
C9H16NO7 - R	C9H17NO7	250.093235	0.026	0.0486 ± 0.0027	0.0347 ± 0.0059	0.0227 ± 0.0025	0.0058 ± 0.0007
C9H14NO7 - R	C9H15NO7	248.077580	0.008	n.d.	0.0042 ± 0.0005	0.0024 ± 0.0003	0.0015 ± 0.0001
C9H12NO7 - R	C9H13NO7	246.061933	0.022	n.d.	n.d.	n.d.	0.0012 ± 0.0001
C9H10NO7 - R	C9H11NO7	244.046283	0.022	n.d.	n.d.	n.d.	0.0010 ± 0.0001
C9H12NO6 - R	C9H13NO6	230.066995	-0.076	n.d.	0.0051 ± 0.0010	0.0048 ± 0.0005	0.0032 ± 0.0002
C9H10NO6 - R	C9H11NO6	228.051337	-0.113	n.d.	0.0059 ± 0.0012	0.0054 ± 0.0002	0.0031 ± 0.0002
C9H8NO6 - R	C9H9NO6	226.035710	-0.013	n.d.	n.d.	n.d.	0.0007 ± 0.0001
C9H10NO5 - R	C9H11NO5	212.056446	-0.011	0.0794 ± 0.0048	0.1106 ± 0.0022	0.0907 ± 0.0044	0.0664 ± 0.0050
C9H8NO5 - R	C9H9NO5	210.040793	-0.026	n.d.	0.0053 ± 0.0004	0.0079 ± 0.0004	0.0064 ± 0.0003
C9H12NO5 - R	C9H13NO5	n.d.	n.d.	n.d.	n.d.	n.d.	n.d.
C9H8NO4 - R	C9H9NO4	194.045881	-0.010	n.d.	0.0071 ± 0.0010	0.0056 ± 0.0003	0.0047 ± 0.0003
C9H10NO4 - R	C9H11NO4	196.061525	-0.041	n.d.	0.0041 ± 0.0003	0.0031 ± 0.0001	0.0030 ± 0.0002
C9H12NO4 - R	C9H13NO4	198.077179	-0.018	n.d.	n.d.	0.0022 ± 0.0002	0.0021 ± 0.0001

Table S2 Ribose-isoleucine derived Maillard reaction products which appear in the "general" Maillard reaction products formation and degradation pathways shown in Fig. 5; (n.d.) not detected or S/N < 8.

				Relative peak intensity (mean ± sd, n = 3)			
**General Formula**	**Ribose-isoleucine**	***m/z* (avg.)**	**error (ppm)**	**2h**	**4h**	**6h**	**10h**
C7H12NO6 - R	C11H21NO6	262.129606	-0.028	0.1929 ± 0.0051	0.1830 ± 0.0019	0.1591 ± 0.0012	0.1304 ± 0.0006
C7H14NO7 - R	C11H23NO7	280.140171	-0.026	0.7262 ± 0.0045	0.6436 ± 0.0035	0.5611 ± 0.0055	0.4913 ± 0.0062
C7H6NO4 - R	C11H15NO4	224.092808	-0.112	n.d.	0.0063 ± 0.0003	0.0099 ± 0.0003	0.0135 ± 0.0003
C7H6NO3 - R	C11H15NO3	208.097909	-0.042	0.0028 ± 0.0003	0.0071 ± 0.0002	0.0124 ± 0.0002	0.0210 ± 0.0011
C7H10NO5 - R	C11H19NO5	244.119044	-0.016	0.0069 ± 0.0005	0.0069 ± 0.0002	0.0063 ± 0.0001	0.0058 ± 0.0001
C7H6NO5 - R	C11H15NO5	240.087744	-0.018	n.d.	0.0021 ± 0.0002	0.0031 ± 0.0002	0.0042 ± 0.0001
C7H8NO4 - R	C11H17NO4	226.108455	-0.126	0.0083 ± 0.0005	0.0075 ± 0.0002	0.0069 ± 0.0001	0.0083 ± 0.0004
C7H10NO6 - R	C11H19NO6	260.113955	-0.031	n.d.	0.0019 ± 0.0000	0.0020 ± 0.0001	0.0015 ± 0.0000
C7H12NO7 - R	C11H21NO7	278.124531	0.010	0.0033 ± 0.0002	0.0045 ± 0.0002	0.0041 ± 0.0002	0.0038 ± 0.0002
C7H8NO5 - R	C11H17NO5	242.103397	-0.002	n.d.	0.0020 ± 0.0002	0.0021 ± 0.0001	0.0020 ± 0.0000
C7H10NO4 - R	C11H19NO4	228.124111	-0.096	n.d.	0.0014 ± 0.0003	0.0031 ± 0.0001	0.0063 ± 0.0003
C7H8NO6 - R	C11H17NO6	258.098316	0.010	n.d.	0.0012 ± 0.0002	0.0011 ± 0.0001	0.0011 ± 0.0001
C7H10NO7 - R	C11H19NO7	276.108884	0.023	n.d.	n.d.	0.0005 ± 0.0000	0.0005 ± 0.0000
C7H12NO5 - R	C11H21NO5	246.134704	0.024	n.d.	n.d.	n.d.	0.0004 ± 0.0000
C7H4NO4 - R	C11H13NO4	222.077157	-0.117	n.d.	n.d.	0.0005 ± 0.0000	0.0007 ± 0.0001
C7H8NO3 - R	C11H17NO3	210.113548	-0.098	n.d.	n.d.	n.d.	0.0004 ± 0.0000
C12H18NO9 - R	C16H27NO9	376.161292	-0.043	0.0032 ± 0.0002	0.0043 ± 0.0003	0.0049 ± 0.0001	0.0054 ± 0.0002
C12H14NO8 - R	C16H23NO8	356.135089	-0.010	n.d.	n.d.	0.0014 ± 0.0001	0.0017 ± 0.0001
C12H16NO8 - R	C16H25NO8	358.150741	-0.006	n.d.	0.0032 ± 0.0000	0.0048 ± 0.0002	0.0067 ± 0.0002
C12H22NO11 - R	C16H31NO11	412.182426	-0.030	n.d.	0.0028 ± 0.0004	0.0026 ± 0.0002	0.0022 ± 0.0000
C12H16NO9 - R	C16H25NO9	374.145641	-0.045	n.d.	0.0011 ± 0.0000	0.0019 ± 0.0001	0.0026 ± 0.0001
C12H20NO10 - R	C16H29NO10	394.171860	-0.033	n.d.	n.d.	0.0011 ± 0.0001	0.0012 ± 0.0000
C12H14NO6 - R	C16H23NO6	324.145264	0.004	n.d.	n.d.	0.0005 ± 0.0002	0.0011 ± 0.0001
C12H12NO6 - R	C16H21NO6	322.129617	0.013	n.d.	0.0016 ± 0.0002	0.0023 ± 0.0002	0.0038 ± 0.0003
C12H12NO7 - R	C16H21NO7	338.124539	0.032	n.d.	n.d.	0.0012 ± 0.0001	0.0014 ± 0.0001
C12H16NO10 - R	C16H25NO10	390.140582	0.024	n.d.	n.d.	n.d.	0.0003 ± 0.0000
C12H16NO7 - R	C16H25NO7	342.155810	-0.053	n.d.	n.d.	0.0004 ± 0.0000	0.0011 ± 0.0001
C12H12NO8 - R	C16H21NO8	354.119417	-0.073	n.d.	n.d.	0.0011 ± 0.0001	0.0016 ± 0.0001
C12H14NO5 - R	C16H23NO5	308.150373	0.080	n.d.	n.d.	n.d.	0.0006 ± 0.0001
C12H20NO9 - R	C16H29NO9	378.176952	-0.017	n.d.	n.d.	0.0011 ± 0.0001	0.0021 ± 0.0002
C12H18NO10 - R	C16H27NO10	392.156230	0.018	n.d.	n.d.	n.d.	0.0003 ± 0.0001
C12H12NO5 - R	C16H21NO5	306.134707	0.029	n.d.	n.d.	0.0007 ± 0.0003	0.0012 ± 0.0001
C12H14NO9 - R	C16H23NO9	372.129979	-0.079	n.d.	n.d.	0.0005 ± 0.0000	0.0006 ± 0.0000
C12H16NO6 - R	C16H25NO6	326.160920	0.021	n.d.	n.d.	0.0004 ± 0.0001	0.0007 ± 0.0001
C12H10NO5 - R	C16H19NO5	304.119057	0.029	n.d.	n.d.	0.0011 ± 0.0000	0.0021 ± 0.0001
C12H10NO6 - R	C16H19NO6	320.113964	0.002	n.d.	n.d.	0.0010 ± 0.0000	0.0017 ± 0.0001
C12H18NO8 - R	C16H27NO8	n.d.	n.d.	n.d.	n.d.	n.d.	n.d.
C10H12NO6 - R	C14H21NO6	n.d.	n.d.	n.d.	n.d.	n.d.	n.d.
C10H12NO5 - R	C14H21NO5	282.134694	-0.015	0.0055 ± 0.0006	0.0077 ± 0.0002	0.0084 ± 0.0002	0.0095 ± 0.0002
C10H10NO5 - R	C14H19NO5	280.119055	0.025	n.d.	0.0010 ± 0.0001	0.0012 ± 0.0001	0.0016 ± 0.0001
C10H14NO7 - R	C14H23NO7	316.140128	-0.158	n.d.	n.d.	0.0008 ± 0.0000	0.0008 ± 0.0000
C10H10NO6 - R	C14H19NO6	n.d.	n.d.	n.d.	n.d.	n.d.	n.d.
C10H16NO9 - R	C14H25NO9	350.145643	-0.042	n.d.	n.d.	0.0006 ± 0.0000	0.0007 ± 0.0001
C10H12NO7 - R	C14H21NO7	n.d.	n.d.	n.d.	n.d.	n.d.	n.d.
C10H14NO5 - R	C14H23NO5	284.150337	-0.040	n.d.	n.d.	0.0005 ± 0.0001	0.0006 ± 0.0000
C10H14NO6 - R	C14H23NO6	300.145273	0.034	n.d.	n.d.	0.0004 ± 0.0000	0.0005 ± 0.0000
C10H16NO8 - R	C14H25NO8	334.150736	-0.020	n.d.	n.d.	0.0008 ± 0.0000	0.0008 ± 0.0001
C10H12NO4 - R	C14H21NO4	266.139770	-0.049	n.d.	n.d.	0.0005 ± 0.0000	0.0008 ± 0.0000
C10H16NO7 - R	C14H25NO7	318.155808	-0.064	n.d.	n.d.	n.d.	0.0003 ± 0.0001
C10H18NO9 - R	C14H27NO9	352.161293	-0.043	n.d.	0.0013 ± 0.0002	0.0009 ± 0.0001	0.0005 ± 0.0000
C10H8NO5 - R	C14H17NO5	278.103412	0.052	n.d.	n.d.	0.0005 ± 0.0000	0.0006 ± 0.0000
C10H8NO6 - R	C14H17NO6	294.098320	0.025	n.d.	0.0011 ± 0.0001	0.0019 ± 0.0001	0.0023 ± 0.0001
C10H18NO8 - R	C14H27NO8	336.166383	-0.030	n.d.	n.d.	0.0009 ± 0.0001	0.0011 ± 0.0001
C10H12NO8 - R	C14H21NO8	330.119453	0.031	n.d.	n.d.	0.0004 ± 0.0001	0.0004 ± 0.0000
C10H16NO6 - R	C14H25NO6	n.d.	n.d.	n.d.	n.d.	n.d.	n.d.
C10H14NO8 - R	C14H23NO8	332.135081	-0.035	n.d.	n.d.	0.0005 ± 0.0001	0.0005 ± 0.0000
C9H16NO8 - R	C13H25NO8	322.150740	-0.009	n.d.	0.0024 ± 0.0001	0.0024 ± 0.0001	0.0022 ± 0.0000
C9H12NO8 - R	C13H21NO8	318.119450	0.022	n.d.	n.d.	n.d.	0.0003 ± 0.0000
C9H14NO8 - R	C13H23NO8	n.d.	n.d.	n.d.	n.d.	n.d.	n.d.
C9H16NO7 - R	C13H25NO7	n.d.	n.d.	n.d.	n.d.	n.d.	n.d.
C9H14NO7 - R	C13H23NO7	304.140185	0.023	n.d.	n.d.	0.0005 ± 0.0000	0.0005 ± 0.0001
C9H12NO7 - R	C13H21NO7	302.124555	0.089	n.d.	n.d.	0.0004 ± 0.0000	0.0004 ± 0.0001
C9H10NO7 - R	C13H19NO7	300.108884	0.020	n.d.	n.d.	0.0011 ± 0.0000	0.0011 ± 0.0001
C9H12NO6 - R	C13H21NO6	286.129613	0.000	n.d.	n.d.	0.0010 ± 0.0001	0.0010 ± 0.0000
C9H10NO6 - R	C13H19NO6	284.113955	-0.027	n.d.	0.0014 ± 0.0002	0.0017 ± 0.0001	0.0018 ± 0.0001
C9H8NO6 - R	C13H17NO6	282.098311	-0.006	n.d.	0.0010 ± 0.0001	0.0012 ± 0.0000	0.0015 ± 0.0001
C9H10NO5 - R	C13H19NO5	268.119055	0.028	0.0231 ± 0.0004	0.0260 ± 0.0007	0.0286 ± 0.0012	0.0293 ± 0.0011
C9H8NO5 - R	C13H17NO5	266.103395	-0.011	n.d.	n.d.	0.0011 ± 0.0000	0.0013 ± 0.0001
C9H12NO5 - R	C13H21NO5	270.134688	-0.036	n.d.	n.d.	0.0006 ± 0.0001	0.0007 ± 0.0001
C9H8NO4 - R	C13H17NO4	250.108485	0.008	n.d.	n.d.	0.0010 ± 0.0001	0.0012 ± 0.0000
C9H10NO4 - R	C13H19NO4	252.124122	-0.044	n.d.	0.0012 ± 0.0002	0.0015 ± 0.0001	0.0017 ± 0.0001
C9H12NO4 - R	C13H21NO4	254.139764	-0.075	n.d.	0.0019 ± 0.0002	0.0030 ± 0.0000	0.0051 ± 0.0002

Table S3 Ribose-lysine derived Maillard reaction products which appear in the "general" Maillard reaction products formation and degradation pathways shown in Fig. 5; (n.d.) not detected or S/N < 8.

				Relative peak intensity (mean ± sd, n = 3)			
**General Formula**	**Ribose-lysine**	***m/z* (avg.)**	**error (ppm)**	**2h**	**4h**	**6h**	**10h**
C7H12NO6 - R	C11H22N2O6	277.140515	0.011	0.6223 ± 0.0038	0.4832 ± 0.0070	0.4049 ± 0.0028	0.3298 ± 0.0013
C7H14NO7 - R	C11H24N2O7	n.d.	n.d.	n.d.	n.d.	n.d.	n.d.
C7H6NO4 - R	C11H16N2O4	239.103728	-0.019	0.0080 ± 0.0004	0.0159 ± 0.0004	0.0219 ± 0.0010	0.0253 ± 0.0020
C7H6NO3 - R	C11H16N2O3	223.108778	-0.177	0.0053 ± 0.0002	0.0088 ± 0.0002	0.0120 ± 0.0002	0.0184 ± 0.0008
C7H10NO5 - R	C11H20N2O5	259.129957	0.037	0.0084 ± 0.0001	0.0070 ± 0.0002	0.0058 ± 0.0000	0.0052 ± 0.0001
C7H6NO5 - R	C11H16N2O5	255.098659	0.045	0.0007 ± 0.0000	0.0013 ± 0.0001	0.0018 ± 0.0001	0.0020 ± 0.0001
C7H8NO4 - R	C11H18N2O4	241.119376	-0.023	0.0051 ± 0.0001	0.0045 ± 0.0001	0.0051 ± 0.0002	0.0094 ± 0.0004
C7H10NO6 - R	C11H20N2O6	275.124882	0.072	0.0037 ± 0.0002	0.0059 ± 0.0001	0.0077 ± 0.0001	0.0072 ± 0.0002
C7H12NO7 - R	C11H22N2O7	293.135435	0.029	0.0008 ± 0.0000	0.0009 ± 0.0000	0.0008 ± 0.0000	0.0008 ± 0.0000
C7H8NO5 - R	C11H18N2O5	257.114308	0.041	0.0030 ± 0.0001	0.0030 ± 0.0000	0.0027 ± 0.0001	0.0022 ± 0.0001
C7H10NO4 - R	C11H20N2O4	243.135033	0.005	0.0008 ± 0.0000	0.0007 ± 0.0000	0.0009 ± 0.0000	0.0014 ± 0.0001
C7H8NO6 - R	C11H18N2O6	273.109228	0.057	0.0012 ± 0.0001	0.0013 ± 0.0000	0.0012 ± 0.0001	0.0009 ± 0.0001
C7H10NO7 - R	C11H20N2O7	291.119781	0.013	0.0012 ± 0.0001	0.0011 ± 0.0000	0.0009 ± 0.0001	0.0005 ± 0.0001
C7H12NO5 - R	C11H22N2O5	261.145613	0.062	0.0003 ± 0.0000	0.0003 ± 0.0000	0.0002 ± 0.0000	0.0003 ± 0.0000
C7H4NO4 - R	C11H14N2O4	237.088069	-0.055	n.d.	n.d.	0.0002 ± 0.0000	0.0001 ± 0.0000
C7H8NO3 - R	C11H18N2O3	225.124433	-0.150	0.0003 ± 0.0000	0.0002 ± 0.0000	0.0002 ± 0.0000	0.0002 ± 0.0000
C12H18NO9 - R	C16H28N2O9	391.172176	-0.080	0.0179 ± 0.0002	0.0255 ± 0.0003	0.0272 ± 0.0006	0.0266 ± 0.0013
C12H14NO8 - R	C16H24N2O8	371.145971	-0.057	0.0026 ± 0.0000	0.0052 ± 0.0001	0.0067 ± 0.0001	0.0072 ± 0.0001
C12H16NO8 - R	C16H26N2O8	373.161623	-0.052	0.0087 ± 0.0002	0.0099 ± 0.0001	0.0091 ± 0.0003	0.0080 ± 0.0005
C12H22NO11 - R	C16H32N2O11	427.193270	-0.157	0.0007 ± 0.0001	0.0006 ± 0.0000	0.0004 ± 0.0000	0.0003 ± 0.0000
C12H16NO9 - R	C16H26N2O9	389.156533	-0.061	0.0012 ± 0.0000	0.0019 ± 0.0000	0.0020 ± 0.0000	0.0019 ± 0.0000
C12H20NO10 - R	C16H30N2O10	409.182703	-0.169	0.0074 ± 0.0015	0.0059 ± 0.0009	0.0044 ± 0.0001	0.0024 ± 0.0000
C12H14NO6 - R	C16H24N2O6	339.156168	0.018	0.0005 ± 0.0000	0.0008 ± 0.0000	0.0009 ± 0.0000	0.0015 ± 0.0001
C12H12NO6 - R	C16H22N2O6	337.140523	0.031	0.0011 ± 0.0000	0.0019 ± 0.0000	0.0025 ± 0.0000	0.0042 ± 0.0002
C12H12NO7 - R	C16H22N2O7	353.135408	-0.054	0.0010 ± 0.0000	0.0021 ± 0.0001	0.0025 ± 0.0000	0.0027 ± 0.0000
C12H16NO10 - R	C16H26N2O10	n.d.	n.d.	n.d.	n.d.	n.d.	n.d.
C12H16NO7 - R	C16H26N2O7	357.166730	0.008	0.0004 ± 0.0001	0.0006 ± 0.0000	0.0006 ± 0.0000	0.0009 ± 0.0001
C12H12NO8 - R	C16H22N2O8	369.130309	-0.090	0.0003 ± 0.0000	0.0006 ± 0.0000	0.0008 ± 0.0000	0.0008 ± 0.0001
C12H14NO5 - R	C16H24N2O5	323.161258	0.033	0.0002 ± 0.0000	0.0003 ± 0.0001	0.0005 ± 0.0001	0.0008 ± 0.0001
C12H20NO9 - R	C16H30N2O9	n.d.	n.d.	n.d.	n.d.	n.d.	n.d.
C12H18NO10 - R	C16H28N2O10	407.167061	-0.150	0.0007 ± 0.0000	0.0009 ± 0.0000	0.0010 ± 0.0000	0.0007 ± 0.0000
C12H12NO5 - R	C16H22N2O5	321.145598	0.004	0.0003 ± 0.0000	0.0006 ± 0.0000	0.0008 ± 0.0000	0.0012 ± 0.0001
C12H14NO9 - R	C16H24N2O9	n.d.	n.d.	n.d.	n.d.	n.d.	n.d.
C12H16NO6 - R	C16H26N2O6	341.171801	-0.031	n.d.	0.0003 ± 0.0000	0.0004 ± 0.0000	0.0006 ± 0.0000
C12H10NO5 - R	C16H20N2O5	355.106619	-0.017	n.d.	0.0003 ± 0.0000	0.0004 ± 0.0000	0.0006 ± 0.0000
C12H10NO6 - R	C16H20N2O6	371.101507	-0.090	n.d.	n.d.	0.0001 ± 0.0000	0.0001 ± 0.0000
C12H18NO8 - R	C16H28N2O8	375.177288	-0.010	n.d.	0.0003 ± 0.0000	0.0004 ± 0.0000	0.0005 ± 0.0000
C10H12NO6 - R	C14H22N2O6	313.140525	0.042	0.0062 ± 0.0001	0.0064 ± 0.0002	0.0059 ± 0.0001	0.0050 ± 0.0001
C10H12NO5 - R	C14H22N2O5	297.145612	0.049	0.0021 ± 0.0000	0.0030 ± 0.0001	0.0034 ± 0.0001	0.0032 ± 0.0000
C10H10NO5 - R	C14H20N2O5	295.129967	0.067	0.0025 ± 0.0001	0.0026 ± 0.0001	0.0027 ± 0.0001	0.0028 ± 0.0001
C10H14NO7 - R	C14H24N2O7	331.151081	0.011	0.0023 ± 0.0000	0.0027 ± 0.0001	0.0026 ± 0.0001	0.0024 ± 0.0001
C10H10NO6 - R	C14H20N2O6	311.124875	0.041	0.0013 ± 0.0001	0.0018 ± 0.0000	0.0017 ± 0.0000	0.0016 ± 0.0001
C10H16NO9 - R	C14H26N2O9	n.d.	n.d.	n.d.	n.d.	n.d.	n.d.
C10H12NO7 - R	C14H22N2O7	329.135423	-0.011	0.0008 ± 0.0000	0.0009 ± 0.0000	0.0009 ± 0.0000	0.0008 ± 0.0001
C10H14NO5 - R	C14H24N2O5	299.161249	0.007	n.d.	n.d.	n.d.	0.0003 ± 0.0000
C10H14NO6 - R	C14H24N2O6	315.156181	0.059	0.0005 ± 0.0000	0.0004 ± 0.0000	0.0004 ± 0.0000	0.0004 ± 0.0000
C10H16NO8 - R	C14H26N2O8	349.161637	-0.015	0.0011 ± 0.0001	0.0010 ± 0.0000	0.0010 ± 0.0000	0.0011 ± 0.0000
C10H12NO4 - R	C14H22N2O4	281.150678	-0.013	n.d.	n.d.	n.d.	0.0003 ± 0.0000
C10H16NO7 - R	C14H26N2O7	333.166731	0.011	0.0005 ± 0.0000	0.0007 ± 0.0000	0.0009 ± 0.0000	0.0009 ± 0.0001
C10H18NO9 - R	C14H28N2O9	n.d.	n.d.	n.d.	n.d.	n.d.	n.d.
C10H8NO5 - R	C14H18N2O5	293.114294	-0.011	0.0003 ± 0.0000	0.0005 ± 0.0001	0.0005 ± 0.0000	0.0006 ± 0.0000
C10H8NO6 - R	C14H18N2O6	309.109220	0.024	0.0003 ± 0.0000	0.0005 ± 0.0000	0.0006 ± 0.0000	0.0005 ± 0.0000
C10H18NO8 - R	C14H28N2O8	n.d.	n.d.	n.d.	n.d.	n.d.	n.d.
C10H12NO8 - R	C14H22N2O8	345.130348	0.016	0.0002 ± 0.0000	0.0003 ± 0.0000	0.0002 ± 0.0000	0.0002 ± 0.0000
C10H16NO6 - R	C14H26N2O6	317.171802	-0.032	0.0002 ± 0.0000	0.0002 ± 0.0000	0.0001 ± 0.0000	0.0001 ± 0.0000
C10H14NO8 - R	C14H24N2O8	347.145978	-0.039	0.0005 ± 0.0001	0.0007 ± 0.0000	0.0007 ± 0.0000	0.0007 ± 0.0000
C9H16NO8 - R	C13H26N2O8	337.161643	0.004	0.0005 ± 0.0001	0.0004 ± 0.0000	0.0004 ± 0.0000	0.0003 ± 0.0000
C9H12NO8 - R	C13H22N2O8	333.130350	0.024	0.0002 ± 0.0000	0.0002 ± 0.0000	0.0002 ± 0.0000	0.0002 ± 0.0000
C9H14NO8 - R	C13H24N2O8	335.145990	-0.006	0.0010 ± 0.0001	0.0017 ± 0.0002	0.0021 ± 0.0001	0.0023 ± 0.0001
C9H16NO7 - R	C13H26N2O7	321.166723	-0.012	0.0049 ± 0.0004	0.0026 ± 0.0002	0.0011 ± 0.0000	0.0003 ± 0.0000
C9H14NO7 - R	C13H24N2O7	319.151081	0.012	0.0014 ± 0.0001	0.0014 ± 0.0001	0.0014 ± 0.0000	0.0013 ± 0.0000
C9H12NO7 - R	C13H22N2O7	317.135434	0.023	0.0006 ± 0.0000	0.0007 ± 0.0000	0.0008 ± 0.0000	0.0007 ± 0.0000
C9H10NO7 - R	C13H20N2O7	n.d.	n.d.	n.d.	n.d.	n.d.	n.d.
C9H12NO6 - R	C13H22N2O6	301.140516	0.014	0.0014 ± 0.0000	0.0015 ± 0.0000	0.0015 ± 0.0000	0.0014 ± 0.0000
C9H10NO6 - R	C13H20N2O6	299.124875	0.043	0.0011 ± 0.0000	0.0015 ± 0.0001	0.0015 ± 0.0001	0.0014 ± 0.0001
C9H8NO6 - R	C13H18N2O6	297.109218	0.021	0.0010 ± 0.0000	0.0012 ± 0.0000	0.0012 ± 0.0000	0.0010 ± 0.0001
C9H10NO5 - R	C13H20N2O5	283.129937	-0.036	0.0896 ± 0.0010	0.0937 ± 0.0018	0.0930 ± 0.0010	0.0842 ± 0.0012
C9H8NO5 - R	C13H18N2O5	281.114299	0.008	0.0011 ± 0.0000	0.0020 ± 0.0000	0.0024 ± 0.0001	0.0027 ± 0.0001
C9H12NO5 - R	C13H22N2O5	285.145590	-0.025	0.0013 ± 0.0000	0.0009 ± 0.0000	0.0009 ± 0.0000	0.0009 ± 0.0000
C9H8NO4 - R	C13H18N2O4	265.119396	0.053	0.0026 ± 0.0000	0.0030 ± 0.0001	0.0030 ± 0.0000	0.0031 ± 0.0001
C9H10NO4 - R	C13H20N2O4	267.135045	0.049	0.0010 ± 0.0000	0.0010 ± 0.0000	0.0010 ± 0.0000	0.0009 ± 0.0000
C9H12NO4 - R	C13H22N2O4	269.150693	0.042	0.0002 ± 0.0000	0.0006 ± 0.0000	0.0008 ± 0.0000	0.0011 ± 0.0001

Table S4 Ribose-cysteine derived Maillard reaction products which appear in the "general" Maillard reaction products formation and degradation pathways shown in Fig. 5; (n.d.) not detected or S/N < 8.

				Relative peak intensity (mean ± sd, n = 3)			
**General Formula**	**Ribose-cysteine**	***m/z* (avg.)**	**error (ppm)**	**2h**	**4h**	**6h**	**10h**
C7H12NO6 - R	C8H15NO6S	252.054714	-0.081	0.5114 ± 0.0106	0.4447 ± 0.0170	0.4223 ± 0.0033	0.3769 ± 0.0069
C7H14NO7 - R	C8H17NO7S	270.065273	-0.095	0.0464 ± 0.0022	0.0390 ± 0.0033	0.0347 ± 0.0008	0.0291 ± 0.0007
C7H6NO4 - R	C8H9NO4S	214.017952	-0.011	0.0002 ± 0.0000	0.0006 ± 0.0001	0.0010 ± 0.0001	0.0023 ± 0.0001
C7H6NO3 - R	C8H9NO3S	198.023031	-0.042	0.0003 ± 0.0000	0.0009 ± 0.0001	0.0019 ± 0.0001	0.0035 ± 0.0001
C7H10NO5 - R	C8H13NO5S	234.044165	-0.017	0.0041 ± 0.0002	0.0080 ± 0.0001	0.0108 ± 0.0010	0.0162 ± 0.0004
C7H6NO5 - R	C8H9NO5S	230.012870	0.004	0.0010 ± 0.0001	0.0025 ± 0.0003	0.0038 ± 0.0006	0.0070 ± 0.0005
C7H8NO4 - R	C8H11NO4S	216.033604	0.001	0.0001 ± 0.0000	0.0004 ± 0.0000	0.0006 ± 0.0001	0.0012 ± 0.0000
C7H10NO6 - R	C8H13NO6S	250.039088	0.016	0.0012 ± 0.0001	0.0021 ± 0.0002	0.0028 ± 0.0002	0.0035 ± 0.0002
C7H12NO7 - R	C8H15NO7S	268.049620	-0.108	0.0039 ± 0.0002	0.0043 ± 0.0004	0.0048 ± 0.0004	0.0058 ± 0.0004
C7H8NO5 - R	C8H11NO5S	232.028520	0.004	0.0013 ± 0.0001	0.0028 ± 0.0001	0.0036 ± 0.0005	0.0051 ± 0.0002
C7H10NO4 - R	C8H13NO4S	218.049256	0.007	n.d.	n.d.	n.d.	0.0003 ± 0.0000
C7H8NO6 - R	C8H11NO6S	248.023439	0.021	0.0004 ± 0.0000	0.0008 ± 0.0000	0.0010 ± 0.0001	0.0014 ± 0.0001
C7H10NO7 - R	C8H13NO7S	n.d.	n.d.	n.d.	n.d.	n.d.	n.d.
C7H12NO5 - R	C8H15NO5S	236.059826	0.031	0.0001 ± 0.0000	0.0001 ± 0.0000	0.0002 ± 0.0000	0.0002 ± 0.0000
C7H4NO4 - R	C8H7NO4S	212.002291	-0.060	n.d.	n.d.	0.0001 ± 0.0000	0.0002 ± 0.0000
C7H8NO3 - R	C8H11NO3S	200.038672	-0.085	n.d.	n.d.	n.d.	0.0001 ± 0.0000
C12H18NO9 - R	C13H21NO9S	366.086426	-0.008	0.0015 ± 0.0001	0.0034 ± 0.0000	0.0055 ± 0.0003	0.0093 ± 0.0002
C12H14NO8 - R	C13H17NO8S	346.060205	-0.026	0.0001 ± 0.0000	0.0001 ± 0.0000	0.0002 ± 0.0000	0.0005 ± 0.0000
C12H16NO8 - R	C13H19NO8S	348.075877	0.037	0.0005 ± 0.0000	0.0012 ± 0.0000	0.0020 ± 0.0002	0.0028 ± 0.0000
C12H22NO11 - R	C13H25NO11S	402.107485	-0.184	0.0137 ± 0.0007	0.0112 ± 0.0009	0.0105 ± 0.0003	0.0097 ± 0.0002
C12H16NO9 - R	C13H19NO9S	364.070775	-0.012	0.0001 ± 0.0000	0.0003 ± 0.0000	0.0004 ± 0.0000	0.0008 ± 0.0001
C12H20NO10 - R	C13H23NO10S	384.096983	-0.029	0.0031 ± 0.0001	0.0061 ± 0.0006	0.0055 ± 0.0002	0.0073 ± 0.0001
C12H14NO6 - R	C13H17NO6S	314.070417	0.104	n.d.	n.d.	n.d.	0.0001 ± 0.0000
C12H12NO6 - R	C13H15NO6S	n.d.	n.d.	n.d.	n.d.	n.d.	n.d.
C12H12NO7 - R	C13H15NO7S	n.d.	n.d.	n.d.	n.d.	n.d.	n.d.
C12H16NO10 - R	C13H19NO10S	380.065698	0.009	0.0001 ± 0.0000	0.0002 ± 0.0000	0.0003 ± 0.0000	0.0004 ± 0.0000
C12H16NO7 - R	C13H19NO7S	332.080988	0.116	n.d.	n.d.	n.d.	0.0002 ± 0.0000
C12H12NO8 - R	C13H15NO8S	344.044613	0.143	n.d.	n.d.	n.d.	0.0001 ± 0.0000
C12H14NO5 - R	C13H17NO5S	n.d.	n.d.	n.d.	n.d.	n.d.	n.d.
C12H20NO9 - R	C13H23NO9S	368.102063	-0.043	n.d.	n.d.	n.d.	0.0001 ± 0.0000
C12H18NO10 - R	C13H21NO10S	382.081338	-0.017	0.0005 ± 0.0000	0.0009 ± 0.0001	0.0012 ± 0.0001	0.0016 ± 0.0001
C12H12NO5 - R	C13H15NO5S	n.d.	n.d.	n.d.	n.d.	n.d.	n.d.
C12H14NO9 - R	C13H17NO9S	362.055128	-0.002	n.d.	n.d.	0.0001 ± 0.0000	0.0002 ± 0.0000
C12H16NO6 - R	C13H19NO6S	n.d.	n.d.	n.d.	n.d.	n.d.	n.d.
C12H10NO5 - R	C13H13NO5S	n.d.	n.d.	n.d.	n.d.	n.d.	n.d.
C12H10NO6 - R	C13H13NO6S	n.d.	n.d.	n.d.	n.d.	n.d.	n.d.
C12H18NO8 - R	C13H21NO8S	350.091536	0.062	n.d.	n.d.	0.0002 ± 0.0000	0.0003 ± 0.0000
C10H12NO6 - R	C11H15NO6S	288.054753	0.067	n.d.	n.d.	n.d.	0.0001 ± 0.0000
C10H12NO5 - R	C11H15NO5S	n.d.	n.d.	n.d.	n.d.	n.d.	n.d.
C10H10NO5 - R	C11H13NO5S	n.d.	n.d.	n.d.	n.d.	n.d.	n.d.
C10H14NO7 - R	C11H17NO7S	306.065316	0.055	0.0001 ± 0.0000	0.0002 ± 0.0000	0.0003 ± 0.0000	0.0005 ± 0.0000
C10H10NO6 - R	C11H13NO6S	286.039127	0.149	n.d.	n.d.	n.d.	0.0001 ± 0.0000
C10H16NO9 - R	C11H19NO9S	340.070787	0.023	0.0052 ± 0.0001	0.0125 ± 0.0007	0.0185 ± 0.0010	0.0289 ± 0.0012
C10H12NO7 - R	C11H15NO7S	304.049673	0.079	n.d.	n.d.	0.0002 ± 0.0000	0.0007 ± 0.0000
C10H14NO5 - R	C11H17NO5S	n.d.	n.d.	n.d.	n.d.	n.d.	n.d.
C10H14NO6 - R	C11H17NO6S	n.d.	n.d.	n.d.	n.d.	n.d.	n.d.
C10H16NO8 - R	C11H19NO8S	324.075874	0.032	n.d.	n.d.	n.d.	0.0006 ± 0.0000
C10H12NO4 - R	C11H15NO4S	n.d.	n.d.	n.d.	n.d.	n.d.	n.d.
C10H16NO7 - R	C11H19NO7S	308.080964	0.048	0.0002 ± 0.0000	0.0002 ± 0.0000	0.0003 ± 0.0000	0.0004 ± 0.0000
C10H18NO9 - R	C11H21NO9S	342.086435	0.017	0.0004 ± 0.0000	0.0006 ± 0.0000	0.0008 ± 0.0000	0.0011 ± 0.0001
C10H8NO5 - R	C11H11NO5S	n.d.	n.d.	n.d.	n.d.	n.d.	n.d.
C10H8NO6 - R	C11H11NO6S	n.d.	n.d.	n.d.	n.d.	n.d.	n.d.
C10H18NO8 - R	C11H21NO8S	326.091529	0.045	0.0002 ± 0.0000	0.0002 ± 0.0000	0.0002 ± 0.0001	0.0001 ± 0.0000
C10H12NO8 - R	C11H15NO8S	n.d.	n.d.	n.d.	n.d.	n.d.	n.d.
C10H16NO6 - R	C11H19NO6S	292.086051	0.059	0.0010 ± 0.0000	0.0011 ± 0.0000	0.0010 ± 0.0001	0.0008 ± 0.0000
C10H14NO8 - R	C11H17NO8S	322.060229	0.047	0.0001 ± 0.0000	0.0003 ± 0.0000	0.0003 ± 0.0000	0.0007 ± 0.0000
C9H16NO8 - R	C10H19NO8S	312.075873	0.029	0.0003 ± 0.0000	0.0005 ± 0.0000	0.0008 ± 0.0001	0.0013 ± 0.0000
C9H12NO8 - R	C10H15NO8S	n.d.	n.d.	n.d.	n.d.	n.d.	n.d.
C9H14NO8 - R	C10H17NO8S	310.060220	0.019	0.0001 ± 0.0000	0.0002 ± 0.0000	0.0002 ± 0.0000	0.0003 ± 0.0000
C9H16NO7 - R	C10H19NO7S	296.080953	0.015	0.0426 ± 0.0017	0.0411 ± 0.0026	0.0372 ± 0.0011	0.0294 ± 0.0007
C9H14NO7 - R	C10H17NO7S	294.065310	0.037	0.0005 ± 0.0000	0.0007 ± 0.0000	0.0008 ± 0.0001	0.0011 ± 0.0000
C9H12NO7 - R	C10H15NO7S	292.049665	0.055	0.0002 ± 0.0000	0.0004 ± 0.0000	0.0004 ± 0.0000	0.0005 ± 0.0000
C9H10NO7 - R	C10H13NO7S	290.034000	0.003	n.d.	n.d.	n.d.	0.0001 ± 0.0000
C9H12NO6 - R	C10H15NO6S	276.054736	0.007	0.0001 ± 0.0000	0.0002 ± 0.0000	0.0003 ± 0.0000	0.0006 ± 0.0000
C9H10NO6 - R	C10H13NO6S	n.d.	n.d.	n.d.	n.d.	n.d.	n.d.
C9H8NO6 - R	C10H11NO6S	n.d.	n.d.	n.d.	n.d.	n.d.	n.d.
C9H10NO5 - R	C10H13NO5S	258.044138	-0.122	n.d.	n.d.	n.d.	0.0002 ± 0.0000
C9H8NO5 - R	C10H11NO5S	256.028480	-0.152	n.d.	n.d.	n.d.	0.0001 ± 0.0000
C9H12NO5 - R	C10H15NO5S	260.059822	0.010	n.d.	n.d.	n.d.	0.0001 ± 0.0000
C9H8NO4 - R	C10H11NO4S	n.d.	n.d.	n.d.	n.d.	n.d.	n.d.
C9H10NO4 - R	C10H13NO4S	n.d.	n.d.	n.d.	n.d.	n.d.	n.d.
C9H12NO4 - R	C10H15NO4S	n.d.	n.d.	n.d.	n.d.	n.d.	n.d.

References (Supporting Information)

1. Lucio, M., Fekete, A., Frommberger, M. & Schmitt-Kopplin, P. in *Handbook of Molecular Microbial Ecology I. Metagenomics and Complementary Approaches,* edited by Frans J. de Bruijn (John Wiley & Sons, Inc2011), pp. 683–695.
2. Moritz, F., Kaling, M., Schnitzler, J.-P. & Schmitt-Kopplin, P. Characterization of poplar metabotypes via mass difference enrichment analysis. *Plant Cell Environ.* **40,** 1057–1073 (2017).
3. Hemmler, D. *et al.* Evolution of Complex Maillard Chemical Reactions, Resolved in Time. *Sci. Rep.* **7,** 3227 (2017).
4. R Development Core Team. *R: A Language and Environment for Statistical Computing. Available at* http://www.R-project.org (Vienna, Austria, 2008).
5. Yaylayan, V.A. Classification of the Maillard reaction: A conceptual approach. *Trends Food Sci. Technol.* **8,** 13–18 (1997).
